# Molecular targets for antifungals in amino acid and protein biosynthetic pathways

**DOI:** 10.1007/s00726-021-03007-6

**Published:** 2021-06-03

**Authors:** Aleksandra Kuplińska, Kamila Rząd

**Affiliations:** Department of Pharmaceutical Technology and Biochemistry and BioTechMed Center, Gdańsk University of Technology, Gdańsk, Poland

**Keywords:** Antifungal targets, Amino acid biosynthesis, Protein biosynthesis, *Candida*, *Aspergillus*, Plant pathogens

## Abstract

Fungi cause death of over 1.5 million people every year, while cutaneous mycoses are among the most common infections in the world. Mycoses vary greatly in severity, there are long-term skin (ringworm), nail or hair infections (tinea capitis), recurrent like vaginal candidiasis or severe, life-threatening systemic, multiorgan infections. In the last few years, increasing importance is attached to the health and economic problems caused by fungal pathogens. There is a growing need for improvement of the availability of antifungal drugs, decreasing their prices and reducing side effects. Searching for novel approaches in this respect, amino acid and protein biosynthesis pathways appear to be competitive. The route that leads from amino acid biosynthesis to protein folding and its activation is rich in enzymes that are descriptive of fungi. Blocking the action of those enzymes often leads to avirulence or growth inhibition. In this review, we want to trace the principal processes of fungi vitality. We present the data of genes encoding enzymes involved in amino acid and protein biosynthesis, potential molecular targets in antifungal chemotherapy, and describe the impact of inhibitors on fungal organisms.

**Supplementary Information:**

The online version contains supplementary material available at 10.1007/s00726-021-03007-6.

## Introduction

Invasive fungal mycoses may affect over 300 million people each year (Stop neglecting fungi 2017; Rodrigues and Nosanchuk 2020) and are responsible for the death of 1.5 million individuals globally (Bongomin et al. 2017), which corresponds to the number of people living in Warsaw, the capital city of Poland. If these estimates are true, it means that in the last 7 months disseminated mycoses caused 875,000 deaths. Skin and nail mycoses affect 20–25% people in the world, which makes them one of the most frequent forms of infection (Havlickova et al. 2008). Fungal diseases are caused by many species, however, 90% of fatal cases result from infections caused by species belonging to the genera of *Aspergillus*, *Candida*, *Cryptococcus* and *Pneumocystis* (Brown et al. 2012). The mortality rate for candidemia is approximately 30–55% and 50–100% for aspergillosis (Brown et al. 2012; Verweij et al. 2016; Haidar and Singh 2018). The most common fungal nosocomial infections are those of endogenous origin, difficult to avoid, because they are caused by opportunistically pathogenic fungi being a part of the human microflora.

Current methods of preventing fungal infections remain unsatisfactory. Indeed, there are several antifungal compounds that are highly effective in some cases; however, they have limitations in use: nephrotoxicity and other adverse effects, drug interactions, and variability in absorption (Quindós et al. 2019). Clinically available antifungals used to treat invasive fungal infections represent four classes of drugs, whereas two of which, polyene macrolide antibiotics and synthetic azole derivatives target ergosterol, a component of the fungal cell membrane, either directly (the former) or its biosynthesis (the latter) (Van den Bossche et al. 1983; Gray et al. 2012). Other targets of established antifungal drugs include β(1 → 3)glucan synthase for echinocandins and RNA biosynthesis for 5-fluorocytosine. In addition, the frequent use of available fungistatic drugs, like Fluconazole, to prevent severe infections in immunocompromised patients, as part of supporting antibiotic therapy or in agriculture, promotes drug resistance, either specific or of the multidrug type (Azevedo et al. 2015). With this in mind, we should mention that right now, there are known cases of fungi resistant to all approved oral drugs (Wiederhold 2017) and the emerging fungal pathogen *Candida auris* is intrinsically resistant to the “golden standard” of antifungal chemotherapy, i.e. Amphotericin B. Depending on the place of research, the observed percentage of frequency of *C. albicans* strains resistant to Fluconazole ranges from less than 1 to 30–40 (Skrodenienė et al. 2006; Gualco et al. 2007; Jafari-Nodoushan et al. 2008). Similarly, azole-resistant *A. fumigatus* strains’ prevalence reached 5% and 6% in the UK and Netherlands, respectively (Snelders et al. 2008; Howard et al. 2009), whereas in one Netherlands hospital, as much as 26% of Aspergillus strains were azole resistant (van Paassen et al. 2016). What is more, late diagnosis as well as the ability of fungal organisms to fast adaptation to a changing environment causes the treatment of mycoses to be excessively difficult and sustained. Therefore, there is an urgent need for the development of novel, highly selective drug candidates with a different mechanism of action for antifungal chemotherapy or for agricultural purposes. Fungal and mammalian cells are highly similar, therefore, to introduce a new antifungal drug, it is desirable to find molecular targets that will be absent in mammalian cells and simultaneously, crucial for the growth and virulence of fungal cells. For these reasons, enzymes unique for fungal cells, involved in amino acids biosynthesis pathways and protein biosynthesis may serve as a great source of molecular targets for novel potential drugs.

This review summarizes current knowledge on potential molecular targets for antifungal chemotherapy or agricultural applications, focusing on enzymes participating in the biosynthetic pathways of human-essential amino acids and involved in protein biosynthesis and post-translational modifications, such as elongation factors and aminoacyl-tRNA synthetases (aaRSa). Examples of numerous inhibitors of these enzymes exhibiting antifungal activity are presented.

## Molecular targets in fungal amino acid biosynthetic pathways

Nine amino acids, namely: l-histidine, l-isoleucine, l-leucine, l-lysine, l-methionine, l-phenylalanine, l-threonine, l-tryptophan and l-valine are regarded as human essential, since there are no pathways of their biosynthesis from simple precursors in human cells. From this perspective, fungal enzymes involved in the pathways of human-essential amino acid biosynthesis might serve as an attractive source of novel molecular targets for antifungal chemotherapy. Yet it might seem questionable whether inhibition of human-essential amino acid biosynthesis would make a successful antifungal treatment, since amino acid requirements may be satisfied by the exogenous supply of amino acid and oligopeptide pool from human serum. From this point of view, the tryptophan and methionine pathways seem especially promising since human serum levels of these amino acids are particularly low enough to disable the rescue of amino acid concentrations caused by inhibition of its biosynthetic pathways in human pathogen cells (Han et al. 2018). Herein, we present the most interesting molecular targets in the fungal amino acid biosynthetic pathway and their potential inhibitors.

### Biosynthesis pathways of amino acids of the aspartate family

**Fig. 1:**
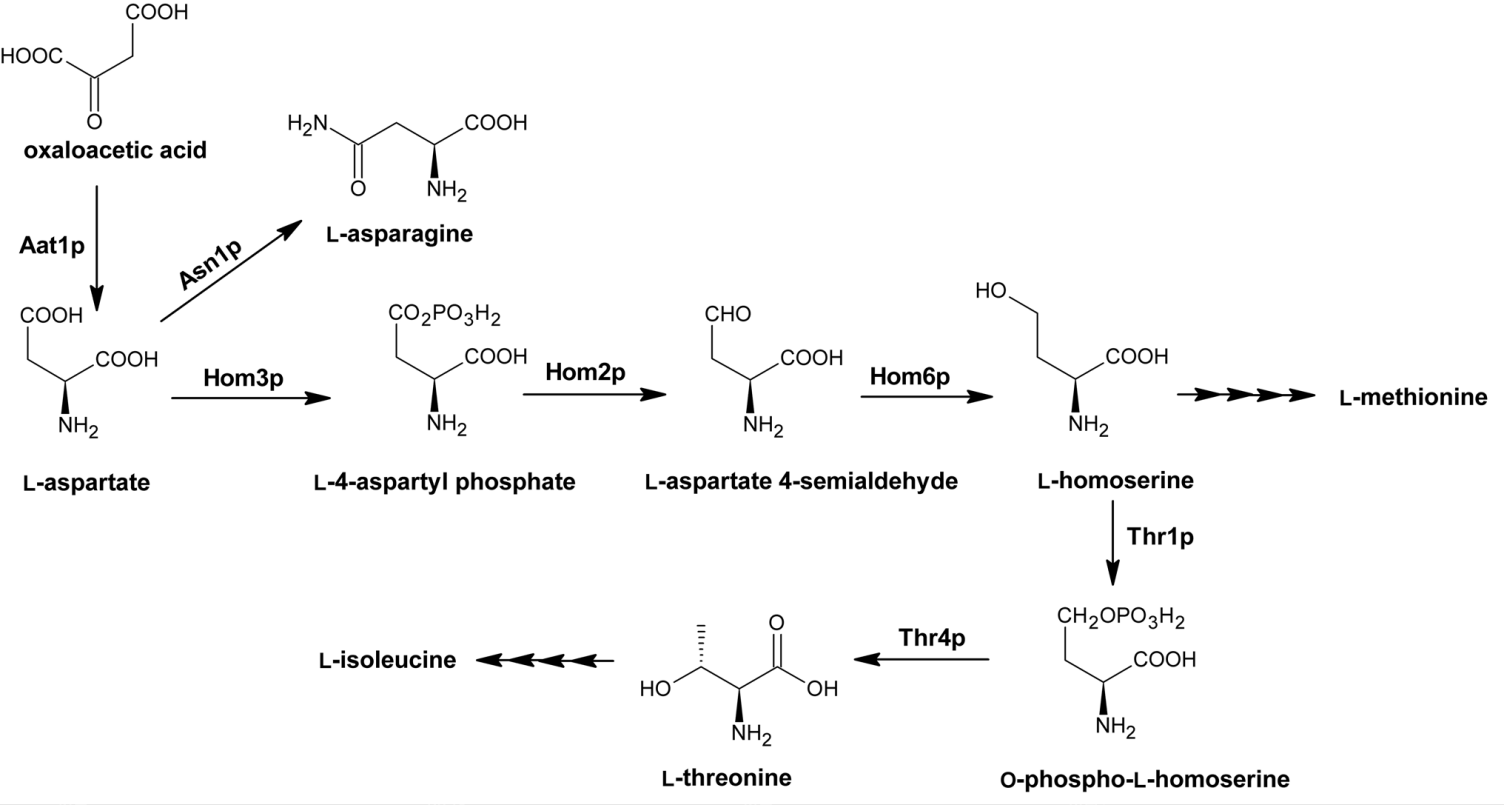
Fungal aspartate family amino acid biosynthesis. Aat1p EC 2.6.1.1 aspartate transaminase; Asn1p EC 6.3.5.4 asparagine synthetase; Hom2p EC 1.2.1.11 aspartate semialdehyde dehydrogenase; Hom3p EC 2.7.2.4 aspartate kinase; Hom6p EC 1.1.1.3 homoserine dehydrogenase; Thr1p EC 2.7.1.39 homoserine kinase; Thr4p EC 4.2.3.1 threonine synthase. Adapted from (Caspi et al. 2018)

Aspartate family refers to amino acids that are synthesized from l-aspartate through pathways absent in mammalian cells (Fig. 1).

Amino acids belonging to the aspartate family are: l-asparagine, l-aspartate, l-threonine, l-methionine, and l-isoleucine. The latter three are human-essential amino acids. Biosynthetic pathways of aspartate family amino acids involve common initial steps that convert oxaloacetate to l-aspartate and next to l-homoserine in three continuous steps. First, l-Aspartate under the action of aspartate kinase (Hom3p) is phosphorylated to l-4-aspartyl phosphate, that in the next step is converted into l-aspartate-4-semialdehyde and then to l-homoserine by aspartate semialdehyde dehydrogenase (Hom2p) and homoserine dehydrogenase (Hom6p). l-Homoserine is the branch point that can lead in two steps to the biosynthesis of either l-threonine and then to l-isoleucine or in several steps to l-methionine (methionine branch). These pathways involve several complex interactions that make them highly investigated in terms of a potential new antifungal target source (Ejim et al. 2004; Kingsbury et al. 2006; Kingsbury and McCusker 2010b).

**Fig. 2:**
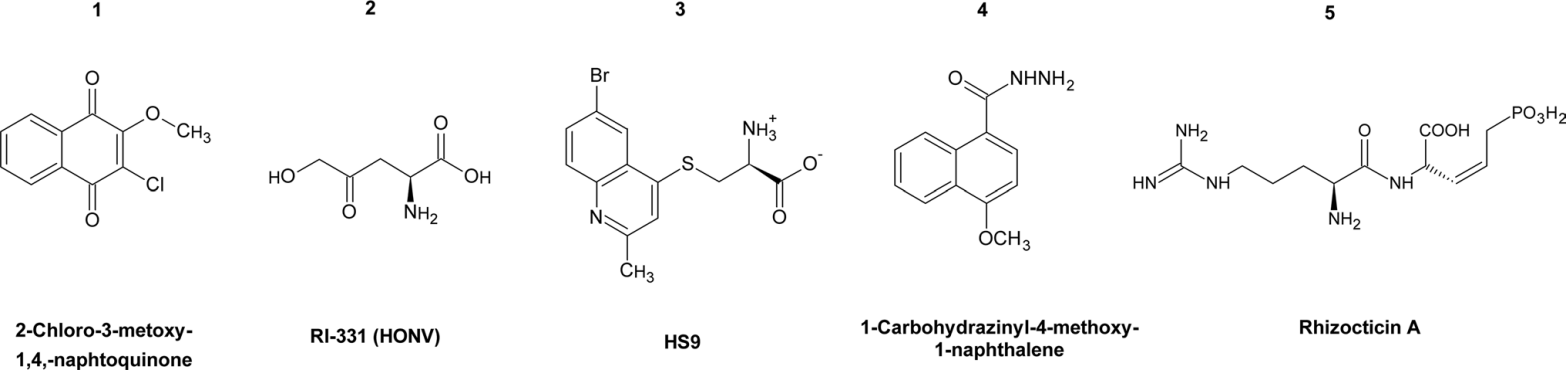
Inhibitors of fungal enzymes from the aspartate family pathway. Compound 1 2-Chloro-3-metoxy-1,4,-naphtoquinone; compound 2 RI-331 (HONV); compound 3 HS9; compound 4 1-Carbohydrazinyl-4-methoxy-1-naphtalene; compound 5 Rhiozocticin A

One of the initial steps in the biosynthesis of aspartate family amino acids is catalyzed by aspartate semialdehyde dehydrogenase encoded by *HOM2* gene. It has been shown that deletion of the gene-encoding Hom2p causes a growth defect of bacteria (Galán et al. 1990; Harb and Kwaik 1998). As far as we know, the growth phenotype of fungal *hom2Δ* mutants has not been studied, however, a recent study provided insights into Hom2p inhibitors derived from *p*-benzoquinone (Dahal and Viola 2018a). Especially one compound, 2-chloro-3-metoxy-1,4-naphtoquinone (Fig. 2, **compound 1**), was effective as an inhibitor of Hom2p in *Cryptococcus neoformans, Candida albicans, Aspergillus fumigatus* and *Blastomyces dermatitidis,* with *K*_*i*_ values ranging from 0.88 to 2.5 µM. Another study determined the first cofactor-bound and inhibitor-bound structures of aspartate semialdehyde dehydrogenase from the pathogenic fungi *B. dermatitidis* (Dahal and Viola 2018b)*.* These structures revealed details of interactions between the enzyme and its inhibitor *p*-benzoquinone that provide insights into the design and development of novel antifungal agents. It is worth mentioning that Viola et al. (2019) invented multiple phthalate derivatives potentially inhibiting aspartate Hom2p present in bacterial cells. The claimed compounds were various derivatives of 4-aminomethylphthalate, but only 3 of them were reported as potent inhibitors of aspartate semialdehyde dehydrogenase from *Streptococcus pneumoniae*.

The next step in the aspartate family amino acid biosynthesis pathway is catalyzed by the homoserine dehydrogenase Hom6p. There have been numerous study reports on the disruption of *HOM6* gene in fungi; *S. cerevisiae hom6Δ* mutants exhibited growth inhibition due to accumulation of toxic aspartate β-semialdehyde and increased sensitivity to the immunosuppressant FK506 (Arévalo-Rodríguez et al. 2004). Disruption of the *HOM6* gene in *C. albicans* caused decreased growth under amino acid starvation that could be rescued by addition of methionine or threonine. Additionally, *HOM6* deletion resulted in decreased cell adhesion, probably because of threonine deficiency that likely leads to a reduction of mannoproteins levels, which are regarded significant for *C. albicans* adhesion and virulence (Kingsbury and McCusker 2008; Tsai et al. 2017). A well-known homoserine dehydrogenase inhibitor is the natural compound known as the antibiotic RI-331 or HONV ((S)-2-amino-4-oxo-5-hydroxypentanoic acid) (Fig. 2, **compound 2**). It was discovered in the 80 s, but due to its antifungal properties, HONV derivatives are still being studied today. HONV was isolated from *Streptomyces* species and found to act as an enzyme-assisted suicide inhibitor of Hom6p, that is effective against *C. albicans, C. tropicalis* and *C. glabrata* but has no effect against *Aspergillus* species (Yamaguchi et al. 1988, 1992; Yamaki et al. 1990; Jacques et al. 2003). It was also proven that HONV can be useful in systematic murine candidiasis treatment in mice (Yamaguchi et al. 1988), however, its effectiveness under physiological conditions should be improved. One of the raised approaches was the eight dipeptides containing HONV as the C-terminal amino acid (Skwarecki et al. 2018). Constructed dipeptides were tested for anticandidal activity in different media. The results showed that HONV dipeptides demonstrated lower antifungal activity than HONV itself in minimal media, however, in RPMI-1640 medium that mimics the composition of low molecular weight compounds in human serum, five dipeptides (Ala-HONV, Ile-HONV, Leu-HONV, Nva-HONV, Val-HONV) demonstrated higher activity than RI-331, with MIC values of 64–128 µg mL^−1^. The HONV-containing oligopeptides seem possible to be further optimized towards enhanced antifungal activity. The more recent study involved a screen of a natural products database towards inhibitors of Hom6p of *Paracoccidioides brasiliensis*, one of the etiological agents of paracoccidioidomycosis (Bueno et al. 2019b). As a result, three molecules were found and tested in vitro*,* resulting in MIC values of 8, 32 and 128 µg mL^−1^. Compound with the lowest MIC/MFC values, HS9 (Fig. 2, **compound 3**), exhibited low cytotoxicity against human cell lines. HS9 is the most active antifungal compound among recently reported in the literature inhibitors of *Paracoccidioides brasiliensis* Hom6p. Summing up, HS9 may be considered as a promising lead compound for further development.

It was reported that *C. albicans hom6Δ* cells were hypersensitive to hygromycin B, thus suggesting Hom6p involvement in protein glycosylation (Tsai et al. 2017), since previously a correlation between defects in protein glycosylation and hygromycin B sensitivity of *S. cerevisiae* was found (Dean 1995). Furthermore, it was revealed by proteomic studies that Hom6p is localized in both cytosolic and cell wall fractions of *C. albicans* (Ebanks et al. 2006; Montserrat Martínez-Gomariz et al. 2009; Tsai et al. 2017). Additionally, novel inhibitors of homoserine dehydrogenase were found among 4-methoxy-naphthalene derivatives (Bagatin et al. 2019). Among the several derivatives, one compound (Fig. 2, **compound 4**) with a carbohydrazide group attached in C1 of 4-methoxy-naphthalene ring was most active against *Paracoccidioides* spp*.* with MIC values ranging from 8 to 32 μg mL^−1^. This compound also showed moderate antifungal activity against *C. albicans, C. parapsilosis* and *C. glabrata* (128–256 μg mL^−1^)*.* What is more, this 4-methoxy-naphthalene derivative and amphotericin B combination resulted in a good synergistic antifungal effect against *P. brasiliensis* and simultaneously did not cause toxicity in monkey kidney and murine macrophage cells.

Conversion of l-homoserine to l-threonine involves two steps catalyzed by homoserine kinase and threonine synthase encoded by *THR1* and *THR4,* respectively. Both of them could be regarded as potential molecular targets for antifungal chemotherapy. It was reported that both *THR1* and *THR4* genes are essential for growth and are required for virulence of *C. albicans* and *C. neoformans* cells (Kingsbury and McCusker 2008, 2010b). It was also suggested that the growth inhibition effect in *THR1* and *THR4* mutants results from toxic homoserine accumulation in yeast cells (Kingsbury and McCusker 2010c). A more recent study revealed that *C. albicans THR1* depleted mutants exhibited increased sensitivity to oxidative and osmotic stress (Lee et al. 2018). Threonine synthase can be inhibited by a natural compound produced by *Bacillus subtilis,* known as Rhizocticin A (L-arginyl-L-2-amino-5-phosphono-3-cis-pentenoic acid**, **Fig. 2, **compound 5**), as reported by Kugler et al. (1990). Rhizocticin A successfully inhibited growth of *S. cerevisiae, Schizosaccharomyces pombe* and *Yarrowia lipolytica* with MIC values of 0.35 μg mL^−1^. No other inhibitors of fungal Thr1p and Thr4p have been reported so far.

### Methionine biosynthesis branch

Every cell needs l-methionine to function properly, most of all, it is needed for protein biosynthesis as it plays a role of its initiator. l-Methionine is also involved in many metabolic processes and works as a precursor for the synthesis of *S*-adenosylomethionine (SAM), needed for the methylation reaction of DNA or phospholipids (Gophna et al. 2005). There are several reports of studies performed on fungal microorganisms confirming that enzymes involved in l-methionine biosynthesis pathway constitute a promising source of targets for new antifungal drugs (Aoki et al. 1995; Pascon et al. 2004).

**Fig. 3:**
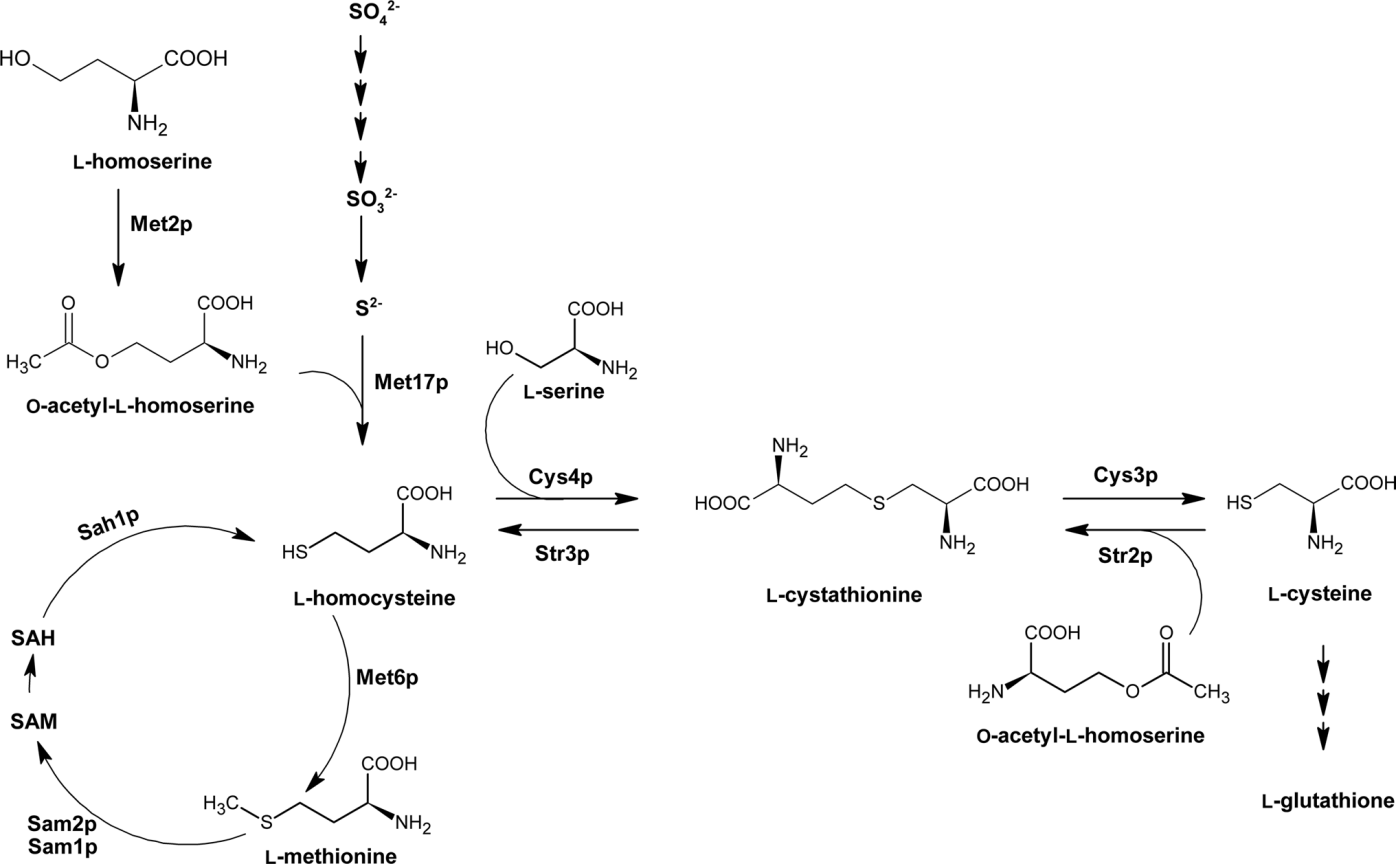
Fungal l-methionine biosynthesis pathway. Cys3p EC 4.4.1.1 cystathionine-γ-lyase; Cys4p EC 4.2.1.22 cystathionine-β-synthase; Met2p EC 2.3.1.31 homoserine O-acetyltransferase; Met6p EC 2.1.1.13 methionine synthase; Met17p EC 2.5.1.49, EC 2.5.1.47 bifunctional O*-*acetyl-l-homoserine/*O*-acetyl-l-serine sulfhydrolase; Sah1p EC 3.3.1.1 adenosylhomocysteinase; Sam1p, Sam2p EC 2.5.1.6 methionine adenosyltransferase; Str2p EC/2.5.1.48 cystathionine-γ-synthase; Str3p EC 4.4.1.8 cystathionine-β-lyase. Adapted from (Caspi et al. 2018)

The methionine branch of l-aspartate amino acid biosynthesis pathway exists in fungal, plant and bacterial cells and starts from l-homoserine, but subsequent reactions are not the same among these organisms (Gophna et al. 2005). In the first step of l-methionine biosynthesis (Fig. 3), in fungal cells, l-homoserine is *O*-acetylated upon the action of homoserine *O*-acetyltransferase (Met2p), however, in plant cells, l-homoserine is *O*-phosphorylated and bacterial cells produce *O*-succinyl-l-homoserine at this step. In the next stage, a sulfur atom is incorporated into the rising amino acid chain, either from inorganic sulfide via direct sulfhydrylation or from l-cysteine through the transsulfurylation pathway (Hébert et al. 2011; Kulikova et al. 2019). In the process of direct sulfhydrylation, *O*-acetyl-l-homoserine sulfhydrolase (Met17p) uses free sulfur atoms and *O*-acetyl-l-homoserine to produce l-homocysteine. Interestingly, in some fungal microorganisms, this step is catalyzed by bifunctional *O*-acetyl-l-homoserine/*O*-acetyl-l-serine sulfhydrolase EC 2.5.1.49, EC 2.5.1.47 (Brzywczy and Paszewski 1993). Introduction of a sulfur atom through the transsulfurylation pathway requires the participation of two enzymes: cystathionine-γ-synthase (Str2p) and cystathionine-β-lyase (Str3p). Str2p utilizes *O*-acetyl-l-homoserine and l-cysteine to produce l-cystathionine, next reaction catalyzed by Str3p yields l-homocysteine. In many organisms, there also exists a reverse transsulfurylation pathway involving l-homocysteine transformation to l-cystathionine by cystathionine-β-synthase (Cys4p) and next its lysis back to l-cysteine upon the action of cystathionine-γ-lyase (Cys3p) (Hébert et al. 2011). The last step of l-methionine biosynthesis involves *S*-methylation of l-homocysteine catalyzed by methionine synthase (Met6p). It can be noted that l-cysteine biosynthesis is strictly related to l-methionine biosynthesis pathway. In some microorganisms, l-cysteine can be produced in two ways: through the reverse transsulfurylation pathway and via* O*-acetylserine pathway (Toh-e et al. 2018; de Melo et al. 2019).

The first enzyme of the l-methionine biosynthesis pathway, homoserine *O*-acetyltransferase (Met2p), is undoubtedly a promising target for novel antifungal agents, since it was found that as required for virulence of *C. neoformans* in a mouse inhalation model (Nazi et al. 2007). Several other studies performed on *C. albicans, C. guilliermondii* and *S. cerevisiae* showed that *MET2* gene-depleted mutant cells are methionine auxotrophs (Singh and Sherman 1974; Kingsbury and McCusker 2010c; Obando Montoya et al. 2014). Nazi et al. (2007) discovered an effective inhibitor of Met2p, however, it was unsuccessful against *C. neoformans* in minimal medium. Since then, few inhibitors of Met2p have been discovered. A study stating high structural similarity between homoserine *O*-transacetylases from different bacterial and fungal species also suggested that the enzyme active site is highly druggable (Chaton et al. 2019). Serine-*O*-acetyltransferase EC 2.3.1.30 (Cys2p) catalyzes similar reaction to that catalyzed by Met2p, but Cys2p uses l-serine as a substrate to produce *O*-acetyl-l-serine instead of *O*-acetyl-l-homoserine. Chen et al. (2019a) found six compounds inhibiting serine-*O*-acetyltransferase from *Staphylococcus aureus* (IC_50_ ranging from 29.83 to 203.13 μM)*.* Two of those, i.e. 11-oxo-ebracteolatanolide B and (4R,4aR)-dihydroxy-3-hydroxymethyl-7,7,10a-trimethyl-2,4,4a,5,6,6a,7,8,9,10,10a,10b-dodecahydrophenanthro[3,2-b]furan–2-one also exhibited antibacterial activity against MRSA with MIC values of 12.5 µg mL^−1^ and 25 µg mL^−1^. In another study, a virtual screening of ChemDiv libraries was conducted to identify potential inhibitors of serine-*O*-acetyltransferase (Magalhães et al. 2020)*.* Out of the 73 evaluated compounds, 6 displayed an inhibitory effect with IC_50_ below 100 μM, but only 1 inhibited the growth of *E. coli* in 20% LB medium (MIC 64 µg mL^−1^). Conclusions resulting from studies on serine-*O*-acetyltransferase inhibitors may be exploited in the rational design of compounds targeting homoserine *O*-acetyltransferase.

Another subsequent enzyme involved in l-methionine biosynthesis pathway is the bifunctional *O*-acetyl-l-homoserine/*O*-acetyl-l-serine sulfhydrolase encoded by *MET17* gene (also named *MET15* and *MET25*). It was shown that *S. cerevisiae* and *C. glabrata* mutants deficient in *O*-acetyl-l-homoserine sulfhydrolase activity were methionine or sulfur auxotrophs (Singh and Sherman 1974; Yadav et al. 2011). These results indicate that *S. cerevisiae* and *C. glabrata* only possess a transsulfurylation pathway allowing for l-cysteine production (Hébert et al. 2011). In addition, it was shown that *S. cerevisiae* cells lacking Met17p activity were more sensitive to sulfometuron methyl agent causing starvation for isoleucine and valine (Bae et al. 2017). Studies performed on *C. guilliermondii* mutant depleted in Met17p encoding gene, revealed its prototrophic character even in the absence of methionine (Obando Montoya et al. 2014). Prototrophic character was also observed while disruption of *O*-acetyl-l-homoserine/*O*-acetyl-l-serine sulfhydrolase encoding gene in *C. albicans* cells, however, it led to a severe defect of growth on sulfate (Viaene et al. 2000). As far as we know, there are no reports of fungal *O*-acetyl-l-homoserine/*O*-acetyl-l-serine sulfhydrolase inhibitors, but there are several examples of inhibitors of the bacterial enzyme (Joshi et al. 2019). The most potent one is 3-((*Z*)-((*Z*)-5-(4-fluorobenzylidene)-3-methyl-4-oxothiazolidin-2-ylidene)amino)benzoic acid, with IC_50_ value of 19 nM (Poyraz et al. 2013).

**Fig. 4:**
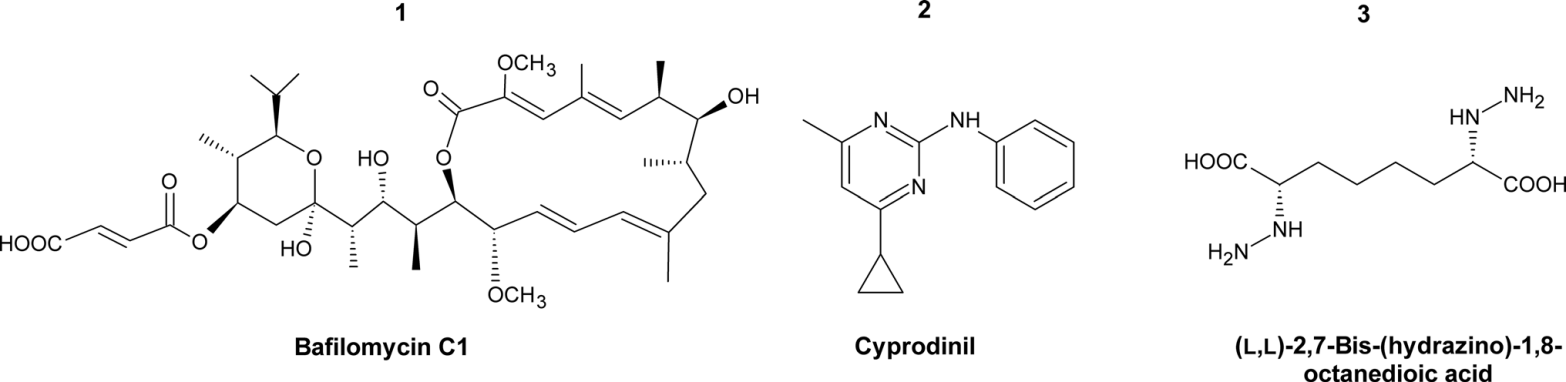
Inhibitors of fungal enzymes from the l-methionine biosynthesis pathway. Compound 1 Bafilomycin C1; compound 2 Cyprodinil; compound 3 (L,L)-2,7,-Bis-(hydrazino)-1,8-octanedioic acid

Methionine synthase is encoded by the *MET6* gene and catalyzes the last step of l-methionine biosynthesis pathway. This enzyme also exists in mammalian cells, but it is structurally different from the fungal version (Ubhi et al. 2014). Mammalian methionine synthase uses cobalamin as a cofactor, while the fungal enzyme is cobalamin independent, resulting in mechanistic differences between both enzyme versions. What is more, inhibition of methionine synthase results in the accumulation of toxic intermediates involved in l-methionine biosynthesis pathway: l-homoserine and l-homocysteine, the former also causes the disruption of ergosterol biosynthesis (Pascon et al. 2004; Kingsbury and McCusker 2010a,b,c). The above reasons raise the possibility that fungal Met6p could be a promising target for the development of novel antifungal drugs. Studies performed with *C. albicans* double deletion mutants of *MET6* gene revealed that Met6p is essential for fungal growth and full virulence in a mouse infection model (Aoki et al. 1995; Suliman et al. 2007). In filamentous fungi, *Aspergillus fumigatus* methionine synthase gene is essential in vivo and required for the invasion of the host cell (Amich et al. 2016). More recent study revealed that *Pichia pastoris MET6* deleted cells were auxotrophic for methionine and adenine, thus indicating that methionine synthase is required for the biosynthesis of both methionine and adenine. Interestingly, this study also showed that Met6p is localized in the nucleus of *P. pastoris* and *C. albicans* cells, but in the cytoplasm of *S. cerevisiae* cells. It was suggested that nuclear localization of Met6p is a unique feature of respiratory yeasts such as *P. pastoris* and *C. albicans* and is essential for the stability and function of the enzyme (Sahu et al. 2017). Surprisingly, up to this date, no successful inhibitors of methionine synthase demonstrating antifungal effect have been reported. Recently, nine bafilomycin compounds were isolated from the fermentation broth of *Streptomyces albolongus* (Ding et al. 2016)*.* Among them, three new compounds were active against *C. parapsilosis* with MIC value 1.56–3.13 μg mL^−1^, but one compound bafilomycin C1 (Fig. 4,** compound 1**), known before as a good antibacterial agent, displayed an antifungal effect towards *C. albicans, C. parapsilosis* and *C. neoformans* with MIC of 1.56 μg mL^−1^. Later, it was discovered that bafilomycin C1 caused a significant downregulation of the expression of ergosterol biosynthesis-related genes in *C. albicans*. Moreover, the expression of methionine synthase encoding gene was also downregulated by 2.7-fold (Su et al. 2018). Met6p is certainly an attractive target for novel antifungal therapies, however, successful inhibitors of methionine synthase still need to be found.

Transsulfurylation pathway utilizes l-cysteine to produce l-homocysteine and involves two enzymes: cystathionine-γ-synthase and cystathionine-β-lyase encoded by *STR2* and *STR3* genes, respectively. In the first step of the transsulfurylation pathway, Str2p uses cysteine and *O*-acetyl-l-homoserine as substrates to produce l-cystathionine, that is transformed in the subsequent reaction to l-homocysteine upon the action of Str3p. *S. cerevisiae* cells depleted in *STR2* and *STR3* genes were unable to grow in a medium containing glutathione as the only sulfur source, and in the case of *S. cerevisiae str3Δ* mutant in a medium containing cystathionine as the sole sulfur source (Hansen and Johannesen 2000). Excess cysteine level can induce a toxic effect, thus its metabolism is important (Kumar et al. 2006). However, a recent study reported that *S. cerevisiae str2Δ* cells unable to use cysteine, did not exhibit significant enhancement of sensitivity to cysteine toxicity, indicating that cysteine is not found as a toxic metabolite in yeast cells (Deshpande et al. 2017). On the other hand, cystathionine-γ-synthase was proven to play an important role in regulating various processes in *Botrytis cinerea* cells (Shao et al. 2016). It was shown that *B. cinerea* cells deprived of *STR2* gene could not grow in a minimal medium, suggesting that Str2p is required for vegetative differentiation of the cells. Deletion mutants exhibited decreased conidation and increased sensitivity to osmotic, oxidative, and thermal stresses. Most importantly, it was shown that *str2Δ* mutants were avirulent on host plant tissue. These results suggest that Str2p takes part in multiple regulatory functions in *B. cinerea*. Since mammalian cells lack the transsulfurylation pathway, Str2p and Str3p should be considered as a potential target for novel antifungal agents. Sagong et al. (Sagong and Kim 2017) determined the crystal structure of cystathionine-γ-synthase from *Corynebacterium glutamicum* complex with its inhibitor dl-(E)-2-amino-5-phosphono-3-pentenoic acid (APPA), but its antifungal activity still needs to be investigated. In a recent study, anilinopyrimidine fungicide derivative Cyprodinil (4-cyclopropyl-6-methyl-*N*-phenylpyrimidin-2-amine) (Fig. 4,** compound 2**) was investigated as a potential inhibitor of enzymes involved in l-methionine biosynthesis pathway (Hou et al. 2018). The results showed that Cyprodinil exhibited strong fungicidal activity against *Sclerotinia sclerotiorum* cells in vitro*,* but it targeted neither cystathionine-γ-synthase (MetB) nor cystathionine-β-lyase (MetC), since the sequence alignment showed that there was no alteration of amino acid in MetB and MetC between cyprodinil-resistant mutants and their sensitive parental strains. There have been few reports of inhibitors targeting enzymes involved in the reverse transsulfurylation pathway: cystathionine-β-synthase (Cys4p) and cystathionine-γ-lyase (Cys3p). A crystal structure of *S. cerevisiae* Cys4p was presented and (L,L)-2,7-bis(hydrazino)-1,8-octanedioic acid (Fig. 4,** compound 3**) has been proposed as its potential inhibitor (Tu et al. 2018)*.* It must be noted that this enzyme-inhibitor modelling study was conducted to bring insights into the development of a therapeutic agent for stroke treatment. In fact, this octanedioic acid derivative has been shown to inhibit purified cystathionine-β-synthase in an animal neuroblastoma model (McCune et al. 2016).

### Branched-chain amino acids biosynthesis

**Fig. 5:**
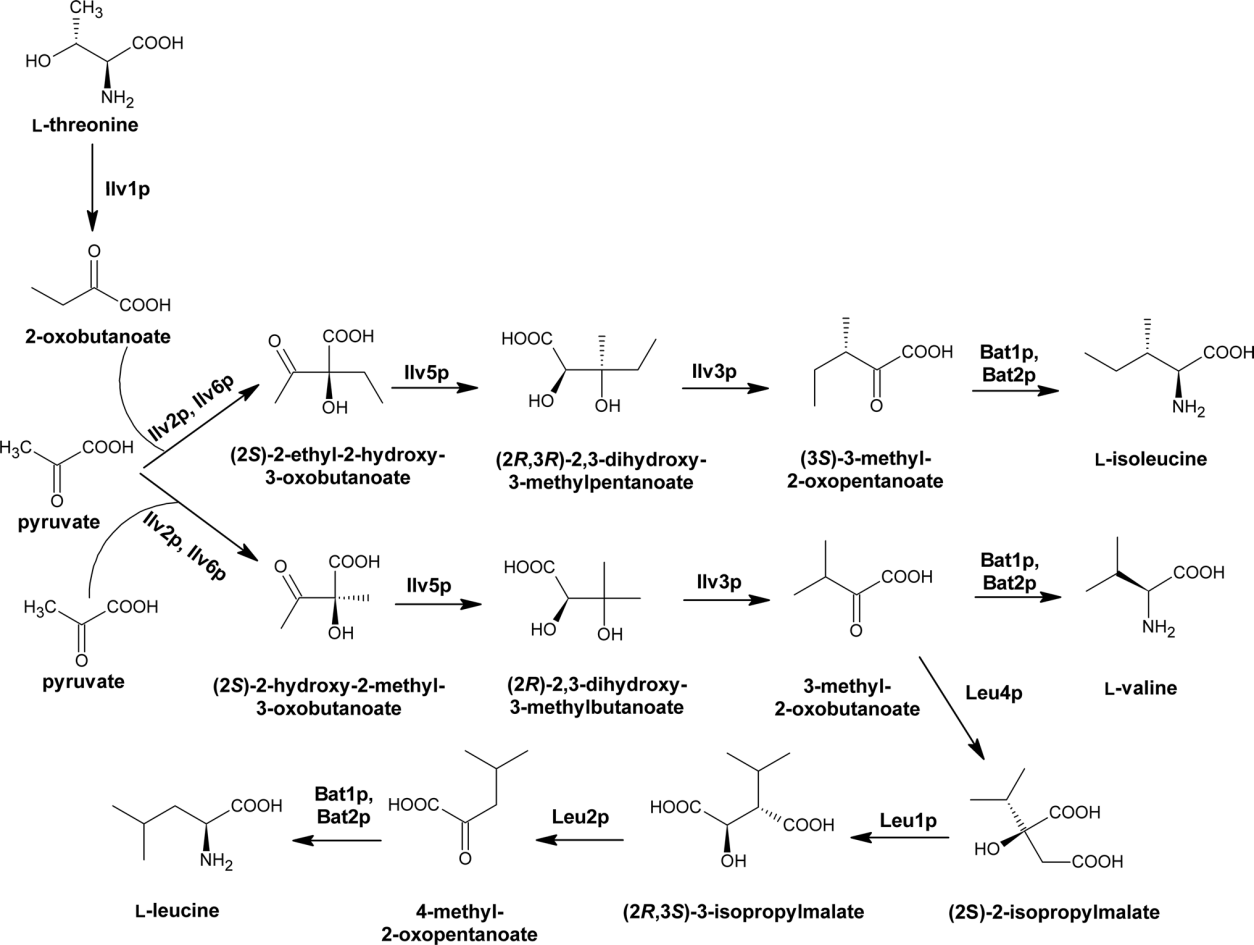
Fungal branched-chain amino acid pathway. Bat1p, Bat2p EC 2.6.1.42 branched-chain amino acid transaminase; Ilv1p EC 4.3.1.19 threonine ammonia-lyase; Ilv2p, Ilv6p EC 2.2.1.6 acetolactate synthase; Ilv3p EC 4.2.1.9 dihydroxyacid dehydratase; Ilv5p EC 1.1.1.86 ketol-acid reductoisomerase (NADP(+)); Leu1p EC 4.2.1.33 3-isopropylmalate dehydratase; Leu2p EC 1.1.1.85 3-isopropylmalate dehydrogenase; Leu4p EC 2.3.3.13 2-isopropylmalate synthase. On the base of (Caspi et al. 2018)

l-Leucine, l-valine and l-isoleucine are amino acids that contain a branched side chain in their structures. These amino acids are essential for humans, because human cells, unlike fungi, are unable of their biosynthesis. This makes enzymes of the branched-chain amino acid fungal pathways as potential targets for antifungal chemotherapy. l-Isoleucine biosynthesis derives from the aspartate pathway and it is parallel with l-valine biosynthesis pathway, since both are catalyzed by the same enzymes, but the intermediates are different (Fig. 5).

l-Isoleucine biosynthetic pathway starts from utilizing l-threonine as a precursor and its conversion into 2-oxobutanoate by the enzyme threonine ammonia-lyase (Ilv1p). In the next step, acetolactate synthase composed of two subunits encoded by *ILV2* and *ILV6* genes, combines pyruvate and 2-oxobutanoate to yield (2S)-2-ethyl-2-hydroxy-3-oxobutanoate. Next reaction is catalysed by ketol-acid reductoisomerase (NADP( +)) encoded by *ILV5* gene, generating (2R,3R)-2,3-dihydroxy-3-methylpentanoate. Subsequently, dihydroxyacid dehydratase (Ilv3p) catalyses the dehydration reaction producing 3-methyl-2-oxobutanoate. Finally, in the last step, branched-chain amino acid transaminase (Bat1p, Bat2p) catalyses the synthesis of l-isoleucine or l-valine. 3-Methyl-2-oxobutanoate, a product of dihydroxyacid dehydratase (Ilv3p) reaction, is a branching point leading either to l-leucine or l-valine biosynthesis. The first committed step of l-leucine biosynthesis is catalyzed by 2-isopropylmalate synthase (Leu4p) utilizing acetyl coenzyme A and 3-methyl-2-oxobutanoate to produce (2S)-2-isopropylmalate. This reaction occurs in mitochondria, from which (2S)-2-isopropylmalate is transported to the cytosol via the oxaloacetate/sulfate carrier Oac1p (López et al. 2015). Two subsequent reactions occur in the cytoplasm and involve 3-isopropylmalate dehydratase (Leu1p) and 3-isopropylmalate dehydrogenase (Leu2p), to catalyze reactions that yield (2R,3S)-3-isopropylmalate and 4-methyl-2-oxopentanoate, respectively. The last step is the branched-chain amino acid transaminase (Bat1p, Bat2p) catalyzed reaction to produce l-leucine (Kohlhaw 2003).

Even though inhibition of l-threonine biosynthesis would simultaneously prevent l-isoleucine biosynthesis, enzymes involved in this pathway are regarded as a potential source of antifungal targets. First, *C. albicans, C. neoformans,* and *S. cerevisiae ilv2*Δ or *ilv1*Δ mutants displayed attenuated virulence in a murine model of infection and revealed a significant loss of viability under isoleucine and valine starvation (Kingsbury et al. 2004, 2006; Pascon et al. 2004; Kingsbury and McCusker 2010a). Moreover, *ILV2* and *ILV6* genes of *Fusarium graminearum* are essential for amino acid biosynthesis, since disruption mutants of both catalytic and regulatory subunits of acetolactate synthase were auxotrophic towards branched-chain amino acids (Liu et al. 2015). Recently, it was discovered that host-induced *ILV2* or *ILV6* gene silencing of one of the most destructive cotton plant fungal pathogen *Verticillium dahlia*, resulted in a dramatic reduction in pathogenicity (Wei et al. 2020).

**Fig. 6:**
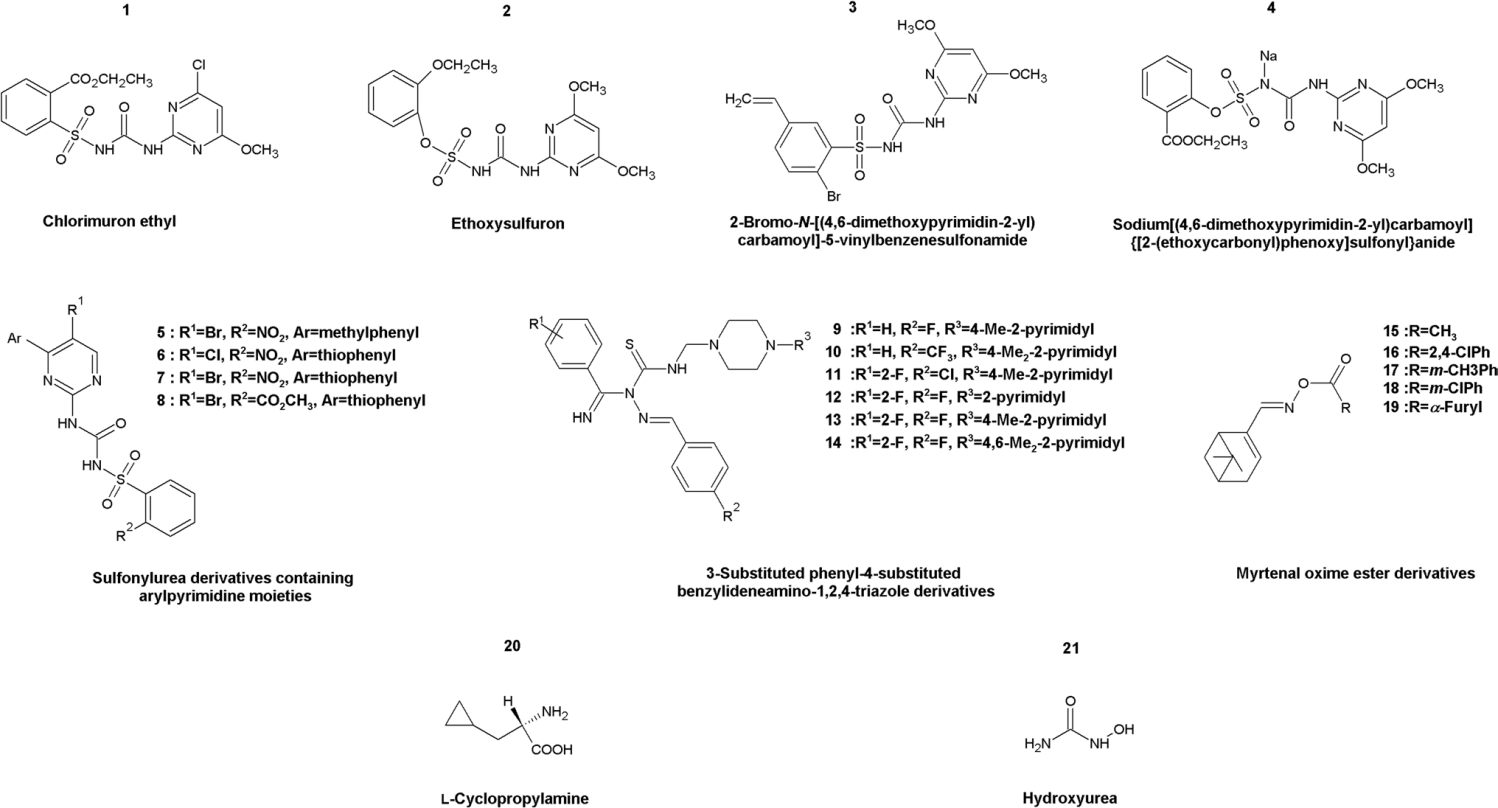
Inhibitors of fungal enzymes from the branched-chain amino acids biosynthesis pathway. Compound 1 Chlorimuron ethyl; compound 2 Ethoxysulfuron; compound 3 2-Bromo-*N*-[(4,6-dimethoxypyrimidin-2-yl)carbamoyl]-5-vinylbenzensulfoamide; compound 4 Sodium[(4,6-dimethoxypyrimidin-2-yl)carbamoyl]{[2-(ethoxycarbonyl)phenoxy]sulfonyl}anide; compounds 5–8 Sulfonylurea derivatives containing arylpyrimidine moieties; compounds 9–14 3-Substituted phenyl-4-substituted benzylideneamino-1,2,4-triazole derivatives; compounds 15–19 Myrtenal oxime ester derivatives; compound 20 l-Cyclopropylamine; compound 21 Hydroxyurea

Until now, several fungal acetolactate synthase inhibitors have been discovered among commercial herbicides belonging to the sulfonylureas family. Especially two well-known herbicides: chlorimuron ethyl and ethoxysulfuron (Fig. 6,** compounds 1–2**) were reported as potent *C. albicans* acetolactate synthase inhibitors, with MIC_50_ value of 2 µM (Lee et al. 2013).

A series of sulfonylurea derivatives containing an alkenyl moiety have been synthesized and said to be putative inhibitors of fungal Ilv2p/Ilv6p, wherein one compound (Fig. 6, **compound 3**) was distinguished to be an active antifungal with ~ 90% in vitro inhibitory rate at 50 μg mL^−1^ dosage in mycelium growth rate method (similarly potent as model compound, chlorothalonil against plant pathogens *Ceratobasidium cornigerum* and *Sclerotinia sclerotiorum*) (Wei et al. 2016). In another study, Garcia et al. (Garcia et al. 2018) discovered that chlorimuron ethyl (Fig. 6,** compound 1**) displayed a broad-spectrum antifungal activity against *C. albicans, C. parapsilosis, C. glabrata* and *S. cerevisiae,* with MIC values of 0.03 μg mL^−1^, 0.003 μg mL^−1^, 0.005 μg mL^−1^, and 0.008 μg mL^−1^, respectively. Antifungal activity of chlorimuron ethyl could be suppressed by supplementation with a combination of isoleucine and valine, but this effect could be abolished upon addition of leucine, proline, or ammonium sulfate. Chlorimuron ethyl turned out to be highly effective in reducing the overall mortality rates of *C. albicans* infected mice by clearing pathogenic fungal burdening of lungs, liver and spleen. In a recent study, 5 novel acetolactate synthase inhibitors have been discovered among 68 derivatives of ethoxysulfuron that displayed increased inhibition of Ilv2p/Ilv6p (Wu et al. 2019)*.* Compound (Fig. 6,** compound 4**) exhibiting the utmost inhibition constant (*K*_*i*_ 6.7 nM) was also the most effective antifungal agent against *C. albicans, C. parapsilosis* and *S. cerevisiae* with MIC values of 1.25 µg mL^−1^, 1.25 µg mL^−1^ and 2.5 µg mL^−1^, respectively. This compound showed activity in the nematode model, suggesting that it could be considered as a candidate for further research. Several novel sulfonylurea inhibitors of acetolactate synthase containing arylpyrimidine moieties have been designed and evaluated for fungicidal activity. Four derivatives (Fig. 6, **compounds 5–8**) showed broad-spectrum and antifungal activity against *Fusarium omysporum, Cercospora arachidicola, Physalospora piricola, Gibberella zeae, Fusarium moniliforme, Sclerotinia sclerotiorum, Corticium gramineum, Helminthosporium maydis, Phytophthora infestans, Rhizoctonia solani* and showed 29–98% in vitro inhibitory rate at 50 μg mL^−1^ concentration (mycelium growth rate method) (Chen et al. 2019b). Above all, these compounds exhibit low toxicity in mammals; therefore, serve as an appealing novel class of antifungals that needs to be further optimized (Garcia et al. 2018).

Ketol-acid reductoisomerase (NADP( +)) is encoded by *ILV5* gene, and turned out to be non-essential for virulence in a murine fungal infection model of *C. albicans* (Becker et al. 2010), nevertheless, there are reports of several inhibitors of this enzyme. First, Wang et al. (Wang et al. 2017) synthesized a series of 3-substituted phenyl-4-substituted benzylideneamino-1,2,4-triazole Mannich bases and bis-Mannich bases that displayed notable activities against *Cercospora arachidicola*, *Physalospora piricola* and *Rhizoctonia cerealis* at the concentration of 50 µg mL^−1^. Especially, several lead compounds (Fig. 6,** compounds 9–13**) appeared fungicidal towards *P. piricola* in the mycelium growth rate test. Interestingly, only one compound (Fig. 6,** compound 14**) that exhibited potent in vitro inhibitory activity against Ilv5p enzyme with K_i_ value of 0.38 µmol L^−1^, displayed a moderate fungicidal activity of 13.6%, 69.6% and 51.4% against *C. arachidicola, P. piricola* and *R. cerealis,* respectively. In another study, compounds targeting ketol-acid reductoisomerase (NADP( +)) were designed and synthesized from a natural forest product α-pinene (Lin et al. 2019); resulting in 18 myrtenal oxime ester compounds that were tested for in vitro antifungal potential towards *Fusarium oxysporum, Fusarium cucumerinum, P. piricola, C. arachidicola, Alternaria solani,* and *Gibberella zeae.* Several compounds (Fig. 6,** compounds 15–19**) showed good antifungal activity against all tested fungi at the concentration of 50 µg mL^−1^ in the agar dilution method; however, the best results were obtained for *P. piricola* with a relative inhibitory rate ranging from 53 to 81%. Recently, the X-ray structure of bacterial ketol-acid reductoisomerase (NADP( +)) from *Staphylococcus aureus* was investigated to model its reaction mechanism and interaction with substrates and inhibitors. In this study, a potential inhibitor, 2-carboxylate-lactic acid, was specially designed based on the indications resulting from the enzyme mechanism. Additionally, two substrate analogues, 2-trihalomethyl acetolactic acid, and 2-glutaryl lactic acid were discovered. The described mechanism-inspired strategy design of enzyme inhibitors could be useful for the exploration of novel antifungal agents (Yu et al. 2020).

Subsequent enzyme in l-isoleucine biosynthesis pathway is dihydroxyacid dehydratase encoded by *ILV3.* This enzyme catalyzes the dehydration reaction producing α-ketoacids, 3-methyl-2-oxobutanoate and (3*S*)-3-methyl-2-oxopentanoate. A recent study demonstrated that deletion of *ILV3* gene of *Fusarium graminearum,* a human pathogenic fungus, is crucial for the biosynthesis of branched-chain amino acids (Liu et al. 2019). *Fgilv3Δ* mutants could not grow on amino acid depleted medium; however, supplementation of exogenous isoleucine or valine rescued the auxotrophy. *ILV3* depleted strains also exhibited reduced virulence, thus indicating that Ilv3p could be an interesting potential antifungal target. It is worth mentioning that product of the reaction catalyzed by dihydroxyacid dehydratase, 3-methyl-2-oxobutanoate, is also involved in pantothenate production, a subsequent precursor of CoA (Meir and Osherov 2018). As far as we know, there are not many successful inhibitors of Ilv3p, however, Yan et al. (2018) discovered that the fungal sesquiterpenoid aspterric acid is an inhibitor of Ilv3p that could be used as a potential herbicide.

The first enzyme of l-leucine biosynthesis pathway, 2-isopropylmalate synthase, is encoded by *LEU4* gene. This enzyme is highly conserved and can be inhibited by leucine via feedback inhibition mechanism (Kohlhaw 2003). It is noteworthy that (2*S*)-2-isopropylmalate formed upon the action of Leu4p, plays a dual role in the cell. It acts as an intermediate for l-leucine biosynthesis and as a coactivator of the Leu3p master regulator. The regulatory protein, Leu3p, modulates the expression of several genes within and beyond amino acid metabolism and senses 2-isopropylmalate concentrations. At high 2-isopropylmalate concentrations, Leu3p acts as an activator of transcription, while at low concentrations, it acts as a repressor (Kohlhaw 2003). In a recent study, Orasch et al. (2019) proved that in *A. fumigatus* deprivation of 3-isopropylmalate dehydratase or 2-isopropylmalate synthase encoding genes (*A. fumigatus* equivalents, *LEUA* and *LEUC*, respectively) results in leucine auxotrophy, that could be cured by exogenous leucine supplementation, with a note that *leuCΔ* mutant required significantly higher supplementation. It was also discovered that lack of LeuAp or LeuCp activity attenuates the virulence of *A. fumigatus* in the *Galleria mellonella* larvae infection model. While all wild-type infected larvae died on day 3, larvae infected with *leuAΔ* or *leuCΔ* mutant strains stayed alive until the end of the experiment (6 days) with survival rates of 60% and 80%, respectively. It was shown that *leuCΔ* mutant was completely avirulent in a mouse infection model. Taken together, this study proved that l-leucine biosynthesis is crucial for the growth and virulence of *A. fumigatus.* 2-Isopropylmalate synthase can also exist in the form of two isoenzymes encoded by *LEU4* and *LEU9* genes, as reported in *S. cerevisiae* (Kohlhaw 2003)*.* It was discovered that *LEU4* and *LEU9 S. cerevisiae* double deletion mutants were absolute leucine auxotrophs in glucose or ethanol containing medium (López et al. 2015). Interestingly, the single *leu9Δ* mutant exhibited wild-type phenotype on glucose or ethanol containing medium as a sole carbon source, whereas the *leu4Δ* mutant was a partial auxotroph (López et al. 2015). Above studies show that 2-isopropylmalate synthase is worth further investigation as a potential molecular target for novel antifungals.

l-Cyclopropylalanine (Fig. 6,** compound 20**) is a natural compound produced by the mushroom *Amanita virgineoides* and exhibits a significant antifungal effect towards 12 fungal species; the most sensitive were *Ascochyta gossypii, Colletotrichum gloeosporioides* and *Fusarium graminearum* (Ma et al. 2017). It was discovered that the addition of leucine, but not any other amino acid, markedly decreased antifungal effect, suggesting that l-cyclopropylalanine inhibits l-leucine biosynthesis pathway. In fact, l-cyclopropylalanine inhibited 2-isopropylmalate synthase purified from *M. tuberculosis,* at the same rate as it was inhibited by leucine. An antifungal effect of L-cyclopropylalanine was tested against *C. albicans, S. cerevisiae,* and *E. coli;* the result showed that l-cyclopropylalanine had a strong antifungal effect with MIC values of 19.2 µM and 44.5 µM towards *C. albicans* and *S. cerevisiae,* respectively. It is important to add that l-cyclopropylalanine shows little (if any) toxicity in rats, thus showing that selectivity towards fungal cells is another of its assets. Taken together, l-cyclopropylalanine should be considered as a lead compound for the future development of novel antifungal agents.

3-Isopropylmalate dehydratase encoded by the *LEU1* gene catalyzes the conversion of (2S)-2-isopropylmalate into (2R,3S)-3-isopropylmalate. In a study performed on *C. neoformans, LEU1* gene deletion cells could not grow in a medium lacking leucine, with ammonium sulfate as a sole nitrogen source, thus confirming that Leu1p is essential for l-leucine biosynthesis (Do et al. 2016). Interestingly, the addition of leucine to the medium containing glutamine or asparagine as the sole nitrogen source restored the growth of *LEU1* mutant cells. This phenomenon was probably due to the nitrogen catabolite repression that interfered with leucine uptake in the presence of ammonium sulfate as a nitrogen source. Further studies revealed that mutant cells depleted in *LEU1* gene possessed dysfunctional mitochondria and were hypersensitive to oxidative stress and cell wall perturbation agents. Most importantly, *leu1Δ* mutants appeared to be attenuated in virulence in a mouse infection model. A more recent study was conducted on *LEU1*-deficient strains of *Magnaporthe oryzae* rice blast fungus (Tang et al. 2019), where deletion of *LEU1* gene also led to leucine auxotrophy that could be rescued by exogenous supplementation of leucine or rice leaf extract. This study also revealed a critical contribution of Leu1p in the pathogenesis, vegetative growth, and asexual development of *M. oryzae*. Results of these studies suggest Leu1p may be a potential target for novel antifungal drugs. Surprisingly, a commonly known drug, hydroxyurea (Fig. 6, **compound 21**), was found to (potentially) affect 3-isopropylmalate dehydratase. Hydroxyurea has an antiproliferative activity that is widely used in the treatment of chronic myeloid leukemia, sickle cell disease, and AIDS. Moreover, it was proven to alter iron–sulfur centers (Fe–S) in vivo via mechanism incorporating the production of reactive oxygen species (ROS) (Huang et al. 2016). Fe–S are metallic cofactors associated with proteins that allow fine redox tuning, and Leu1p happens to contain one. The study revealed hydroxyurea altered Leu1p Fe–S centers in *S. cerevisiae* cells, but not in the purified enzyme. Up to 3 h exposure of the wild-type yeast strains to hydroxyurea resulted in a three to fourfold decrease in Leu1p activity. However, hydroxyurea did not alter semi-purified Leu1p activity, even at higher concentrations. This suggests that the deleterious effect of hydroxyurea on Fe–S centers might be due to cell metabolism (Huang et al. 2016). Nevertheless, this interesting aspect of altering Leu1p activity requires further studies.

### Biosynthesis of aromatic amino acids

**Fig. 7:**
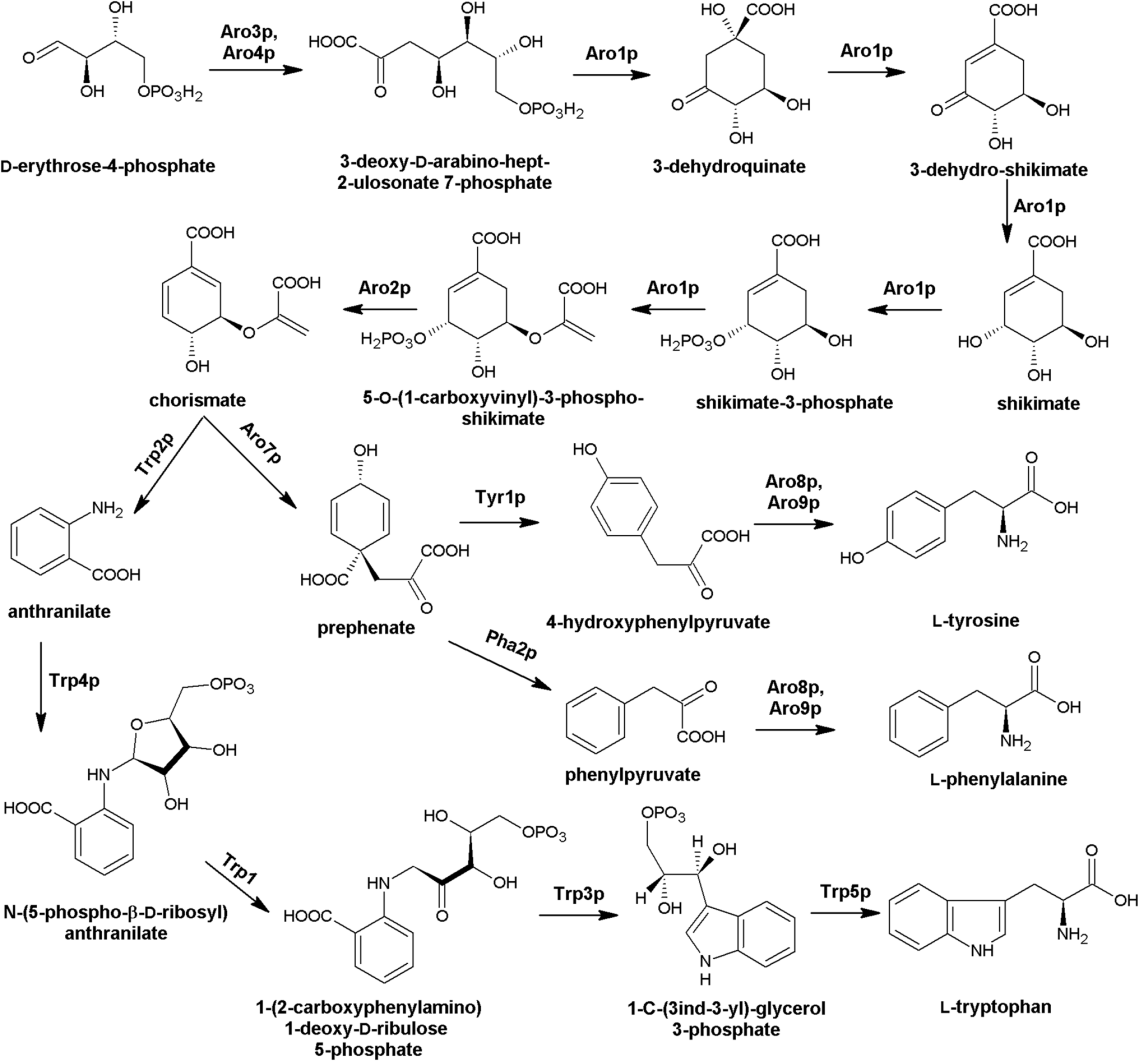
Fungal shikimate pathway. Aro1p EC 2.5.1.19, EC 4.2.1.10, EC 4.2.3.4, EC 1.1.1.25, EC 2.7.1.71 pentafunctional AROM polypeptide; Aro2p EC 4.2.3.5 chorismate synthase; Aro3p, Aro4p EC 2.5.1.54 3-deoxy-7-phosphoheptulonate synthase; Aro7p EC 5.4.99.5 chorismate mutase; Aro8p, Aro9p EC 2.6.1.57, EC 2.6.1.39 aromatic amino acid/2-aminoadipate transaminase; Pha2p EC 4.2.1.51 prephenate dehydratase; Trp1p EC 5.3.1.24 phosphoribosylanthranilate isomerase; Trp2p EC 4.1.3.27 anthranilate synthase; Trp3p EC 4.1.1.48 indole-3-glycerol-phosphate synthase; Trp4p EC 2.4.2.18 anthranilate phosphoribosyltransferase; Trp5p EC 4.2.1.20 tryptophan synthase; Tyr1p EC 1.3.1.13 prephenate dehydrogenase (NADP(+)). On the base of (Caspi et al. 2018)

Bacteria, plants, and fungi synthesize aromatic amino acids, that is, l-phenylalanine, l-tyrosine, and l-tryptophan, throughout the so-called shikimate pathway, because shikimate serves as a major intermediate (Mir et al. 2015). In contrast, mammals must acquire l-Phe and l-Trp from their diet (the enzyme converting l-Phe into l-Tyr is present in mammals), since they are essential for primary metabolism. The shikimate pathway converts d-erythrose-4-phosphate and phosphophenylopyruvate to d-chorismate in seven enzymatic steps catalyzed by: 3-deoxy-7-phosphoheptulonate synthase (Aro3p/Aro4p**),** pentafunctional AROM polypeptide (Aro1p) and chorismate synthase (Aro2p) (Braus 1991) (Fig. 7). d-Chorismate serves as a last common precursor for the synthesis of a variety of aromatic compounds, including aromatic amino acids. Enzyme chorismate mutase (Aro7p) converts D-chorismate to prephenate, an intermediate for l-tyrosine and l-phenylalanine biosynthesis. However, the intermediate for l-tryptophan biosynthesis is anthranilate, produced from d-chorismate by anthranilate synthase (Trp2p). The aromatic amino acid biosynthesis pathway has been intensively studied in plants and bacteria, yet it has been proven that bacteria lacking any gene involved in the shikimate pathway cannot survive in vivo (Mir et al. 2015). Unfortunately, the fungal biosynthetic pathway of aromatic amino acids is poorly categorized. Nevertheless, enzymes of the shikimate pathway serve as an attractive source of molecular targets for the design of novel antifungal drugs. In fact, there are already several reports on the vital role of fungal enzymes involved in the biosynthesis of aromatic amino acids (Sousa et al. 2002; Brunke et al. 2010).

**Fig. 8:**
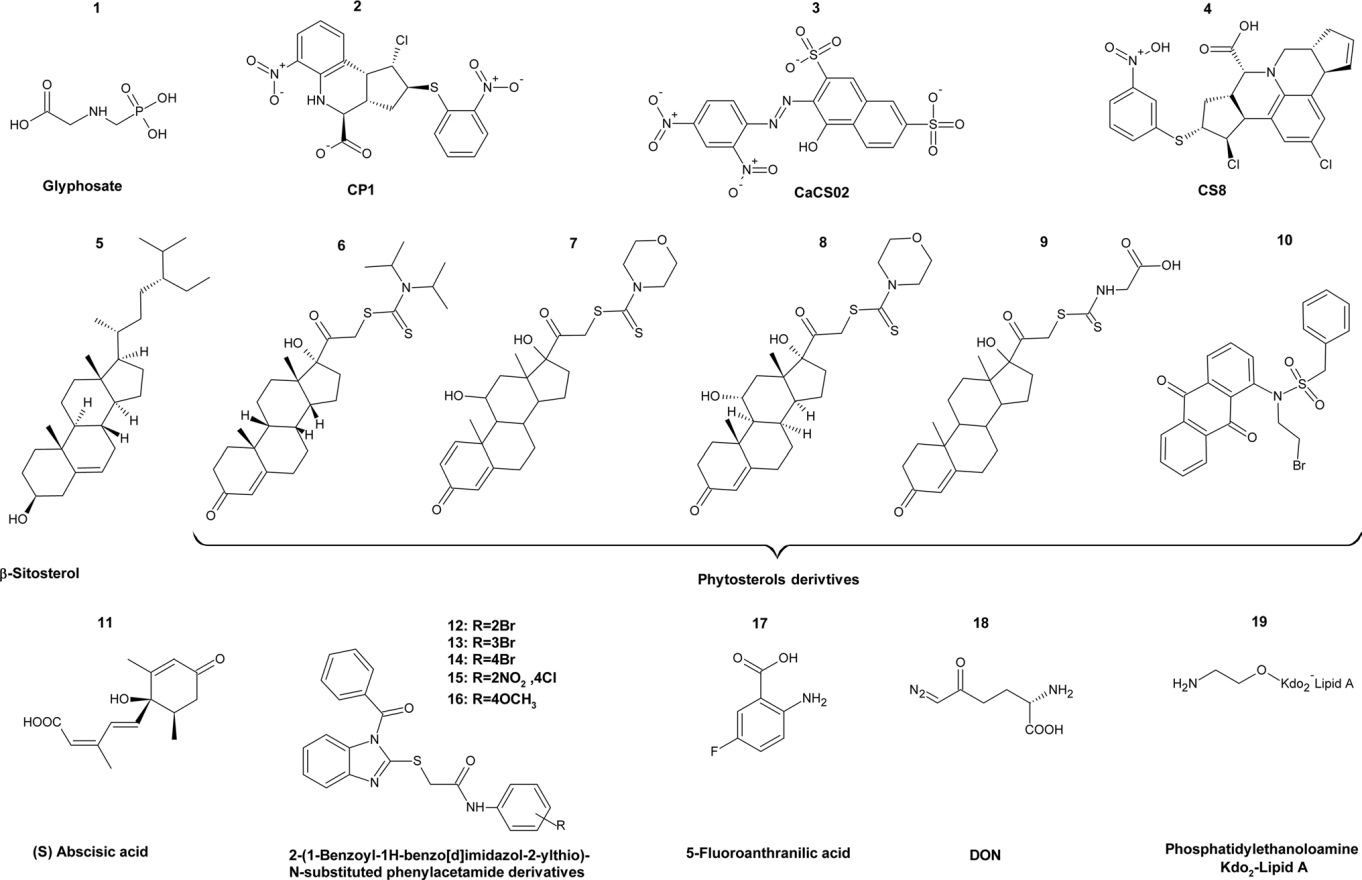
Inhibitors of fungal enzymes from the shikimate pathway. Compound 1 Glyphosate; compound 2 CP1; compound 3 CaCS02; compound 4 CS8; compound 5 β-Sitosterol; compounds 6–10 Phytosterols derivatives; compound 11 (S) Abscisic acid; compounds 12–16 2-(1-Benzoyl-1H-benzo[d]imidazol-2-ylthio)-*N*-substituted phenylacetamide derivatives; compound 17 5-Fluoroanthranilic acid; compound 18 DON; compound 19 Phospatidylethanoloamine Kdo2-Lipid A

One of the first enzymes appearing in the fungal aromatic amino acid biosynthesis pathway is the pentafunctional AROM polypeptide encoded by *ARO1* gene. This enzyme is particularly interesting because of its complex, multi-domain structure that allows catalyzing five consecutive steps of the shikimate pathway (Duncan et al. 1988). There are not many reports on fungal Aro1p investigation, however, via the doxycycline-repressible expression system, the *ARO1* gene was defined as essential for *C. albicans* (O’Meara et al. 2015). Recently, Yeh et al. (2018) provided new insights. In this study, *C. albicans* tetracycline-regulated (TR) knockdown strains depleted in *ARO1* gene displayed decreased growth without aromatic amino acids supplementation and changes in the cell wall chemical composition and architecture. Moreover, it was shown that mutant strains exhibited attenuated virulence in *Galleria mellonella* larvae infection model. In another study, a *C. albicans* mutant strain was constructed, in which one *ARO1* gene allele was deleted and replaced by the *ARG4* selection marker and the second allele was inactivated using poly-adenosine insertion method (Tournu et al. 2019). One study revealed that the deletion strain could not grow in any standard growth medium, yet the growth could be restored with supplementation of 10 mM aromatic amino acids as the sole nitrogen source, therefore, it was concluded that *ARO1* gene is not essential in *C. albicans.* No successful inhibitors of fungal Aro1p are known, however, a popular herbicide glyphosate (Fig. 8,** compound 1**) inhibits the plant 3-phosphoshikimate 1-carboxyvinyltransferase EC 2.5.1.19, that is said to be an orthologue of yeast Aro1p (Rong-Mullins et al. 2017). A study conducted on various *S. cerevisiae* strains showed that glyphosate indeed affected the growth of yeast strains, but not all at the same rate (Rong-Mullins et al. 2017). Additionally, *ARO1* gene-deficient mutants were constructed to investigate whether glyphosate targets yeast Aro1p. Interestingly, most mutant strains were viable, except for *S. cerevisiae* RM11, a wine yard isolate. Surprisingly enough, *ARO1* deficient mutant cells grown in YM medium with glyphosate, showed no difference in growth rate compared to the wild type. The study also tested whether supplementation of tyrosine, tryptophan, or phenylalanine would affect *aro1Δ* yeasts growth rate in the presence of glyphosate. It was revealed that *S. cerevisiae* S288c *aro1Δ* (a laboratory strain) were more sensitive to glyphosate than the wild type, while *S. cerevisiae* YJM789 *aro1Δ* (clinical isolate) mutant was more resistant to glyphosate than the wild type. It was later concluded that the growth variation of tested yeasts was not due to polymorphisms within *ARO1*, but it was rather caused by genetic variation in the amino acid permease and ABC multiple drug transporter. Nonetheless, glyphosate not only inhibits Aro1p, but probably also other non-canonical targets, but that inquiry requires further investigation.

A branch point enzyme of the shikimate pathway, chorismate synthase, is encoded by *ARO2* gene. Singh et al. (2010) identified and characterized *ARO2* gene in *Verticillium longisporum* fungal plant pathogen, using a novel RNA-mediated gene silencing method. Reduction of the expression of both Aro2p encoding isogenes (*ARO2-1, ARO2-2*) by 80% resulted in a bradytrophic character, that is, a leaky auxotrophic mutant for aromatic amino acids. Constructed strains exhibited significantly reduced virulence towards the plant host *Brassica napus* compared to the wild type. Recently, with the help of *S. cerevisiae* chorismate synthase structure as a template, homology modeling of three-dimensional structure of *Paracoccidioides brasiliensis* enzyme was made (Rodrigues-Vendramini et al. 2019). This enabled a virtual screening search for potential inhibitors and molecular dynamics that resulted in one best hit compound CP1 (Fig. 8, **compound 2**). In vitro analysis of CP1 compound revealed that it binds to chorismate synthase and inhibits its activity with IC_50_ of 47 µM. What is more, CP1 displayed antifungal activity towards *Paracoccidioides* spp. with MIC values of 2–16 µg mL^−1^ and 16–32 µg mL^−1^ towards *P. brasiliensis* and *P. lutzii,* respectively. In vivo studies on a mouse infection model proved CP1 compound was highly effective in treating paracoccidioidomycosis*,* by reducing fungal burden in the lungs and inflammatory response. It is important to note that CP1 exhibited no cytotoxic effect on human cell lines. Taken together, CP1 seems to be a promising antifungal agent that should be considered as a lead compound for the development of novel chorismate synthase inhibitors. Not long after, the amino acid sequence of *C. albicans* Aro2p was used for modeling the enzyme’s 3D structure and a large-scale virtual screening (Bueno et al. 2019a). As a result, two novel potential inhibitors of chorismate synthase were identified, CaCS02 and CS8 (Fig. 8, **compounds 3–4**), where the last one shows high resemblance to CP1 structure. Antifungal in vitro activity of both compounds against *C. albicans* and *P. brasiliensis* strains was determined. MIC values of compounds CaCS02 and CS8 towards *P. brasiliensis* were 32 µg mL^−1^ and 512 µg mL^−1^, respectively, however, no antifungal activity against *C. albicans* was observed. CaCS02 inhibited the activity of *P. brasiliensis* Aro2p with IC_50_ value of 29 µM. Similarly, to CP1, CaCS02 appeared not cytotoxic to mammalian cell lines, even more it displayed a synergistic effect with amphotericin B, thus demonstrating potential activity in a combination antifungal therapy. Recently, another 3D modeling study of Aro2p structure from *Alternaria arborescens* plant pathogen was conducted (Choudhary et al. 2020). Natural product database screening identified 2861 compounds as potential inhibitors of *A. arborescens* chorismate synthase. Further computer-aided analysis selected five most potent compounds with a cyclopentaphenanthrene ring backbone (Fig. 8** compounds 6–10**), similar to the one found in plant phytosterols: β-sitosterol (Fig. 8** compound 5**). All identified inhibitors displayed selectivity towards pathogen’s Aro2p, but only five compounds were used for further analysis. Molecular dynamics of enzyme-inhibitor complexes showed that only chorismate synthase-β-sitosterol complex acquired stability. Moreover, β-sitosterol displayed a significant reduction of *A. arborescens* growth in in vivo antifungal bioassay. This study shows that natural β-sitosterol could be useful in taming fungal pathogens in eco-friendly manner.

Chorismate mutase is encoded by the *ARO7* gene and is the enzyme catalyzing the first committed step that leads to the biosynthesis of l-phenylalanine and l-tyrosine, dividing it from the l-tryptophan biosynthesis branch. Aro7p catalyzes prephenate formation from chorismate. Recently, the chorismate mutase activity was described in *Trichoderma parareesei*, a filamentous fungus working as a biofungicide by protecting plants against pathogens. Successful plant colonization by *Trichoderma* spp*.* is considered being a major cause of its beneficial effects (Pérez et al. 2015). Silencing of *T. parareesei ARO7* gene was evaluated in in vitro plant assays. It was reported that mutant cells exhibited a reduced ability to colonize plant roots. Additionally, plants colonized by *ARO7* silenced mutants were more sensitive to *B. cinerea* infection and displayed significantly lower growth parameters. It was, therefore, concluded that chorismate mutase might be involved in the biocontrol potential of *T. parareesei* and is required for maintaining balanced interactions with plants. Fungal Aro7p still needs to be investigated. A homology modeling study was performed for *C. albicans, C. parapsilosis, A. niger,* and *T. rubrum* putative chorismate mutase structures, to enable rational drug design for potential Aro7p inhibitors that could work as antifungal agents (Khedr et al. 2018). Previously, it was discovered that endo-oxabicyclic and aza-bicyclic compounds act as transition state inhibitors of chorismate mutase, and thus were used for the development of a pharmacophore model for virtual screening analysis (Sträter et al. 1997; Hediger 2004; Khedr et al. 2018). Screen of 160.00 lead-like compounds resulted in the identification of 25 compounds that fulfilled Lipinski’s rule of five and exhibited the lowest RMSD values. Two compounds were used for structure similarity search of a potential Aro7p inhibitor that would be easy to synthesize; (S) abscisic acid (Fig. 8, **compound 11**) sheared 98% similarity and was chosen for further analysis. Abscisic acid is a natural sesquiterpene phytohormone that regulates plant growth and development and plays an important role in plant responses to stresses. (S) Abscisic acid’s ability to inhibit Aro7p was tested via inhibition of phenylpyruvate production; the results suggested that indeed (S) abscisic acid targets chorismate mutase. However, the inhibition rate of abscisic acid was compared to Aro7p inhibition by its natural allosteric inhibitor–tyrosine, and it was shown that tyrosine is 2–3 times more potent than (S) abscisic acid. Nonetheless, in vitro analysis of the antifungal properties of (S) abscisic acid revealed antifungal activity against *C. parapsilosis* and *T. rubrum* (MIC 62.5 µg mL^−1^) and *C. albicans* and *A. niger* (MIC 125 µg mL^−1^). Several potential inhibitors of Aro7p were found among 2-(1-benzoyl-1H-benzo[d]imidazol-2-ylthio)-*N*-substituted acetamides derivatives (Yadav et al. 2018). Four derivatives (Fig. 8,** compounds 12–15**) displayed excellent antifungal properties with MIC values ranging from 0.013 to 0.027 µg mL^−1^ against *C. albicans* and *A. niger,* for comparison fluconazole minimal inhibitory concentration is 0.47 µg mL^−1^. These compounds were also effective against several bacteria species such as *S. aureus, B. cereus, B. subtillis, S. typhi,* and *E. coli* (MIC 0.027 µg mL^−1^). All the 20 found derivatives were tested for chorismate mutase inhibition activity, yet only 1 derivative (Fig. 8,** compound 16**) was proven to moderately inhibit mycobacterial Aro7p by 58.23%. It is still unclear whether 2-(1-benzoyl-1H-benzo[d]imidazol-2-ylthio)-N-substituted acetamide derivatives work as fungal Aro7p inhibitors.

L-Tryptophan biosynthesis starts at the branch point, i.e., chorismate, and involves five unique steps catalyzed by anthranilate synthase (Trp2p), anthranilate phosphoribosyltransferase (Trp4p), phosphoribosylanthranilate isomerase (Trp1p), indole-3-glycerol-phosphate synthase (Trp3p) and lastly tryptophan synthase (Trp5p). All these enzymes have been characterized in *C. neoformans* cells and interestingly, *S. cerevisiae* phosphoribosylanthranilate isomerase encoded by *TRP1* gene, in *C. neoformans* cells is encoded by *TRP3* gene. Even more, it was reported that L-tryptophan biosynthetic pathway is essential in *C. neoformans,* as revealed by iRNA silencing of *TRP3* and *TRP5* genes (Fernandes et al. 2015). The study showed that *TRP3* mutants were lethal when grown in YNB medium with galactose and ammonium sulfate as a sole nitrogen source, whereas *TRP5* mutant cells exhibited significantly reduced growth. For both mutants, growth could not be rescued by supplementation of tryptophan. The same phenomenon was discovered for *TRP3* silenced mutants when grown on YNB medium with galactose and proline as the only nitrogen source, however, in this case, growth could be slightly improved with tryptophan supplementation. The *TRP5* mutant could also uptake tryptophan in the proline presence. Notably, both *TRP3* and *TRP5 C. neoformans* mutants remained vital in YNB medium with dextrose, regardless of nitrogen source or tryptophan addition. It was concluded that the growth improvement caused by the use of proline as the sole nitrogen source was probably due to more efficient intracellular tryptophan transport. Moreover, l-tryptophan biosynthesis pathway was a target of an antimetabolite 5-fluoroanthranilic acid (Fig. 8,** compound 17**), since it caused complete cell growth inhibition in YNB medium supplemented with tryptophan. 5-Fluoroanthranilic acid serves as a substrate for Trp4p and disturbs cell growth by generating toxic tryptophan analogues. Additionally, a minimum inhibitory concentration assay was conducted for a glutamine analogue, 6-diazo-5-oxo-l-norleucine (DON) (Fig. 8,** compound 18**) targeting Trp2p. DON inhibits *C. neoformans* growth at the µmolar scale in rich medium, with MICs of 62.5 µM and 125 µM depending on the strain (Fernandes et al. 2015). Recently, *Aspergillus fumigatus* genome has been evaluated and as a result, five essential genes were identified among which there was a *TRP5* gene-encoding tryptophan synthase (Srivastava et al. 2018). By means of homology modelling, Trp5p structure was obtained and then screened against a small molecule pathway database. Further docking studies identified one potential *A. fumigatus* tryptophan synthase inhibitor: phosphatidylethanolamine kdo_2_-lipid A (Fig. 8,** compound 19**). However, up to now, there are no reports on in vitro or in vivo assays of this compound as a potential antifungal agent.

## Protein biosynthesis

Protein biosynthesis is essential for pathogen proliferation within the human host, and it involves the cooperation of enzymes catalyzing the processes of transcription, translation, and post-translational processing. Enzymes involved in the above processes are investigated as potential targets for novel antifungal therapies, in fact a few have already been proposed as targets for antibacterial, antiparasitic and antifungal drugs (Liu et al. 2018). Herein, we would like to focus on fungal protein biosynthesis inhibitors targeting aminoacyl-tRNA synthetases (aaRSs), elongation factors, *N*-myristoyltransferases (NMTs) and enzymes involved in post-translational modifications.

## Aminoacyl-tRNA synthetases

**Fig. 9:**
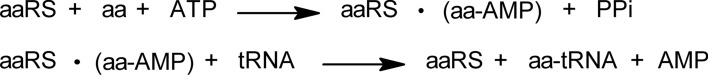
Reactions catalyzed by aaRSs. aa Amino acid; (aa-AMP) Enzyme-bound aminoacyl-adenylate intermediate; aaRS aminoacyl-tRNA synthetases; aa-tRNA aminoacyl-tRNA; AMP adenosine monophosphate; ATP adenosine triphosphate; PPi inorganic pyrophosphate; tRNA transfer RNA

The fundamental process of all living organisms, protein biosynthesis is carried by ribosomes that translate the information encoded in the messenger RNA (mRNA) to the amino acid sequence of the synthesized protein. Ribosomes link individual amino acids with the help of cognate transfer RNAs (tRNAs) and specific aminoacyl-tRNA synthetases (aaRSs) EC 6.1.1. Aminoacyl-tRNA synthetases (aaRSs) exist in all organisms and are essential for protein biosynthesis. AaRSs fulfill two important roles in protein translation: they mediate the condensation of the correct amino acids and its homologous tRNAs, but also they are the only enzymes responsible for ensuring the fidelity of translation. Up to now, 23 different aaRSs have been described which are named according to the aminoacyl-tRNA product generated; one for each of the 20 proteinogenic amino acids, with an exception for lysine that has 2 corresponding aaRSs, and 2 other pyrrolysyl-tRNA synthetase EC 6.1.1.26 and *O*-phosphoseryl-tRNA synthetase EC 6.1.1.27 found only in some bacteria and archaea (Rubio Gomez and Ibba 2020). Condensation of the tRNA molecule with the corresponding amino acid catalyzed by aaRSs involves two-step reaction (Fig. 9). In the first step, aaRSs utilizes ATP molecule to form enzyme-bound aminoacyl–adenylate intermediate, and the subsequent second step involves transfer of the aminoacyl group to the 3′ end of the homologous tRNA.

**Fig. 10:**
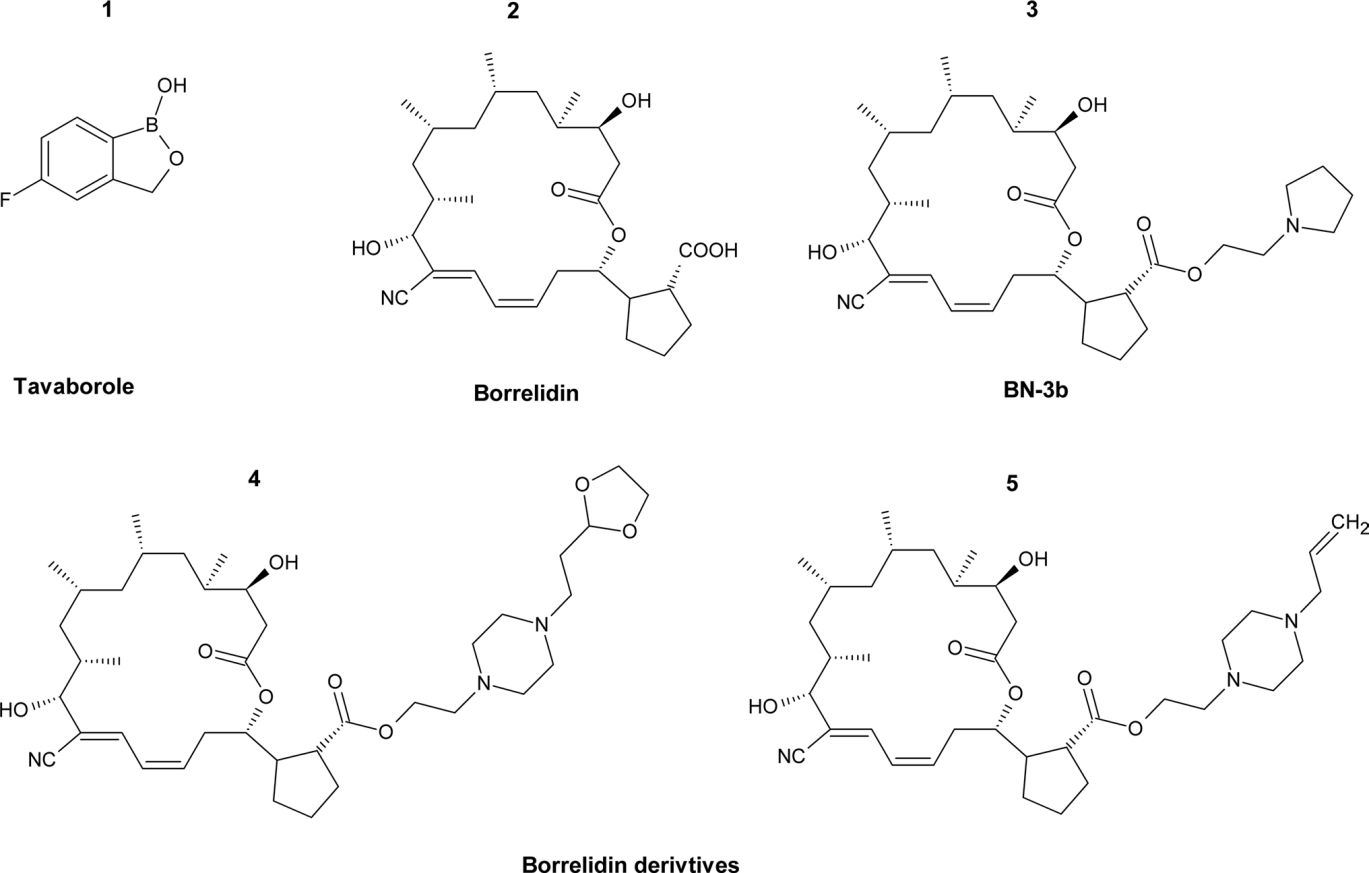
Inhibitors of fungal aminoacyl-tRNA synthetases. Compound 1 Tavaborole; compound 2 Borrelidin; compound 3 BN-3b; compound 4–5 Borrelidin derivatives

Due to their major role in protein biosynthesis, aminoacyl-tRNA synthetases are regarded as potential targets for novel antifungal agents, despite the existing homology between aaRSs from different microorganisms. In fact, several inhibitors of bacterial and fungal aaRSs have been reported (Zhang and Ma 2019; Rubio Gomez and Ibba 2020). Among these inhibitors, cispentacin is worth mentioning, since it was effective against *C. albicans* in a mouse infection model (Oki et al. 1989). Its more potent derivative icofungipen, also known as BAY 10-888, reached phase II of clinical trials; despite exhibiting good clinical efficacy, its research has been discontinued due to side effects observed in human toxicity studies (McCarthy and Walsh 2018). AN2690, a derivative of borinic acid quinolone ester, known as tavaborole (Fig. 10,** compound 1**), is an inhibitor of leucyl-tRNA^Leu^ synthase EC 6.1.1.4 (LeuRS; aaRS charges tRNA^Leu^ with leucine) and is used in clinical treatment since 2014 (Rock et al. 2007; Markham 2014; Sharma and Sharma 2015). Tavaborole displays good antifungal activity against dermatophytes, *Trichophyton mentagrophytes,* and *Trichophyton rubrum*, primary cause of onychomycosis, with MICs values of 1–2 µg mL^−1^ (Coronado et al. 2015). It is also active against *A. fumigatus, Candida* spp., and *S. cerevisiae* (MICs 0.25–4 µg mL^−1^), however, because of its short half-life span, it can only be used as a treatment for topical fungal infections of the nail (Gupta and Versteeg 2016). On the other hand, a recent study reported that tavaborole antifungal activity against molds and yeast from onychomycosis is lower than stated previously (MIC 4–16 µg mL^−1^) (Abastabar et al. 2018). This suggests that this drug might not be the best candidate for the treatment of onychomycosis caused by *Candida* spp*.*, *Aspergillus* spp., and dermatophytes. AN2690 was also found to inhibit LeuRS of *Leishmania*, whereas its derivative ZCL039 was active against *S. pneumonia* with MIC value of 5 µg mL^−1^ (Hu et al. 2013; Manhas et al. 2018).

Another example of aaRS inhibitor is borrelidin (Fig. 10,** compound 2**), a nitrile containing 18-membered polyketide macrolide that inhibits threonyl-tRNA^Thr^ synthetase EC 6.1.1.3 (ThrRS; aaRS charge tRNA^Thr^ with threonine). This compound has a broad-spectrum activity against viruses, bacteria, and some fungi (Lumb et al. 1965; Gao et al. 2012). Despite its promising properties, borrelidin is not used in clinical practice because of its strong cytoxic effect in animal cells, thus suggesting that ThrRS might not be the only target of borrelidin (Wilkinson et al. 2006). A recent study synthesized a library of 47 borrelidin derivatives and evaluated their antifungal activity and cytotoxicity (Hu et al. 2018). The results indicated one compound, BN-3b (Fig. 10,** compound 3**) that was effective against *C. albicans* and *C. parapsilosis* with MIC values of 50 µg mL^−1^ and 12.5 µg mL^−1^, respectively. Two other derivatives (Fig. 10,** compounds 4–5**) had an inhibitory effect on *A. fumigatus* with MIC = 12.5 µg mL^−1^. All tested strains were unaffected by borrelidin at a high concentration of 100 µg mL^−1^. Structure–activity relationship analysis revealed that the cyano group is essential for antifungal activity that can be improved by introduction of (–OCH_2_CH_2_N–) fragment into the carboxyl of borrelidin structure. Molecular docking analysis with X-ray structure of yeast mitochondrial ThrRS showed that the introduced carboxyl substituents could favor extra interaction with ThrRS of pathogenic fungi. Above derivatives displayed far better selectivity towards fungal cells than the parent compound. Later on, the same research group further investigated BN-3b derivative and revealed that it stimulated endogenous reactive oxygen species (ROS) accumulation, leading to an antifungal effect (Su et al. 2020). Additionally, BN-3b severely damaged *C. albicans* cell membrane and inhibited hyphal formation. Moreover, in vivo studies showed that BN-3b significantly prolonged survival and decreased fungal burden in a mouse model of disseminated candidiasis. These findings reveal that induction of endogenous ROS plays a major role in the antifungal activity of BN-3b. However, previously revealed interaction of BN-3b with ThrRS might also be important for antifungal activity, yet it needs to be further verified. Nonetheless, BN-3b should be regarded as a promising lead for the development of novel antifungal agents.

### Elongation factors

Protein biosynthesis occurs via the translational machinery, in which tRNA interacts with the ribosome in three stages: initiation, elongation, and termination. During the elongation stage, a new amino acid is added to the growing polypeptide chain via a three-step process involving selection of an appropriate aminoacyl-tRNA, formation of a peptide bond and finally translocation. Translocation is a crucial step in the process of protein biosynthesis, incorporating elongation factors associated with GTP hydrolysis (Berg et al. 2015). Elongation factors are potential molecular targets for novel antifungal agents not only because of their essential role in protein biosynthesis but also because of exploitable differences between mammalian and fungal counterparts. Fungal translation is unique since it requires three factors: elongation factor 1 (eEF1A), elongation factor 2 (eEF2), and elongation factor 3 (eEF3). In mammalian cells, eEF2 is functionally distinct from the fungal counterpart, while eEF3 does not exist (Dever et al. 2016).

**Fig. 11:**
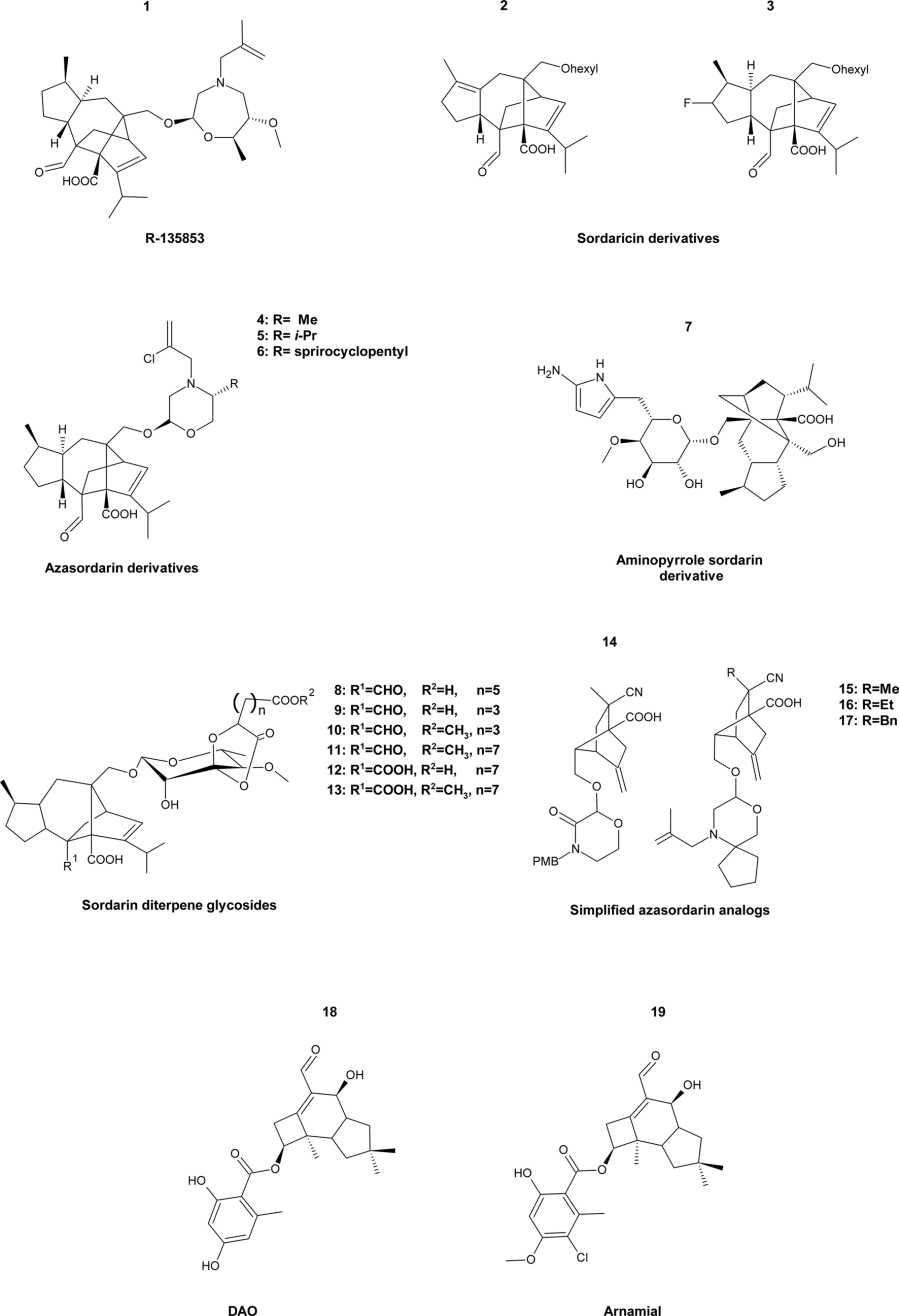
Inhibitors of fungal elongation factors. Compound 1 R-135853; compounds 2–3 Sordaricin derivatives; compounds 4–6 Azasordarin derivatives; compound 7 Aminopyrrole sordarin derivative; compounds 8–13 Sordarin diterpene glycosides; compounds 14–17 Simplified azasordarin analogs; compound 18 DAO; compound 19 Arnamial

Sordarins are protein biosynthesis inhibitors that work in a dual way. They bind to eEF2 and block translocation by stabilization of the eEF2-ribosome complex, but also inhibit eEF2-ATP mediated ribosome splitting (Chakraborty et al. 2013). It is noteworthy that eEF2 are highly conserved among all eukaryotes, however, sordarins interact specifically with eEF2 in yeast and filamentous fungi while failing to do the same in human cells. Several sordarin derivatives exhibiting a broad spectrum of antifungal activity have already been reported (Di Santo 2008). In particular, compound R-135853 (Fig. 11,** compound 1**), a derivative of sordarin isolated from *Zopfiella marina,* was a potent antifungal agent against *C. albicans*, *C. glabrata*, *C. guilliermondii* and *C. neoformans* with MICs in the 0.016–0.5 μg mL^−1^ range. Additionally, R-135853 was effective in *C. albicans* infected mice model (Kamai et al. 2005). Regueiro-Ren et al. (Regueiro-Ren et al. 2002) prepared core-modified sordaricin (aglycone of sordarins) derivatives via biotransformation of *Nocardia* spp. All new compounds exhibit antifungal activity. The most potent two (Fig. 11, **compounds 2–3**) of them inhibit 90% growth of *C. albicans* and *C. glabrata* at 0.125–8 μg mL^−1^ concentration. A year after, Serrano-Wu et al. (Serrano-Wu et al. 2003) synthesized 5′- and 5′-6′-substituted azasordarin derivatives, which were the first sordarins active against *Aspergillus* spp.. 5′-Me analogues (Fig. 11,** compounds 4–5**) were more active against *Candida* spp., *C. neoformans,* and *Aspergillus* spp. than 6′-Me counterparts and registered MIC values < 0.06–32 μg mL^−1^. Substitution of 5′-position broadened the antifungal spectrum of the azasordarins. 5′ Spirocyclopentyl-analogue (Fig. 11,** compound 6**) exhibited the best MIC profile < 0.008–8 μg mL^−1^, while *i*-Pr substitution in the 5′-position improved the compound’s metabolic stability and plasma half-life compared to other azasordarins. A computer-aided design study indicated sordarin aminopyrrole derivative (Fig. 11,** compound 7**) as a potential fungicidal agent, which is ineffective against human eEF2 (Chakraborty et al. 2016). Introduction of 2-aminopyrrole group at C61 position might increase the binding affinity with fungal eEF2 and simultaneously decrease interactions with the human eEF2 cavity, suggesting 2-aminopyrole as a good template for the design of novel antifungal drugs. Another study discovered six novel sordarins from *Curvularia hawaiiensis* TA26-15, a marine-derived fungus, possessing a rare in nature sordarose residue with a spiro 1,3-dioxolan-4-one ring (Zhang et al. 2019). Isolated compounds (Fig. 11, **compounds 8–13**) were tested for antifungal activity and displayed MIC values in the range of 2.9–13 μM against *C. albicans.* Further analysis indicated that the C2 carboxylic acid determined antifungal activity, however, aliphatic acid side chain length may contribute to this effect. Wu and Dockendorff (2018) designed novel scaffolds of sordarin derivatives that could be more easily modified to improve properties such as metabolic stability and antifungal activity. Generated novel scaffolds maintained the pharmacophore of sordarin, which is a carboxylic acid at C1 and a nitrile or aldehyde group at C2 of the bicyclic core, additionally, a complex diterpene core was replaced with a simplified bicyclic scaffold. Docking analysis revealed that simplified sordarin analogs exhibited comparable docking poses to the parent sordarin and similar docking scores to compounds that have been reported to be effective against *S. cerevisiae*. These findings may lead to the design of a soradrin derivative with improved properties. Not long after, the same research group synthesized a series of new simplified azasordarin analogs with bicyclo[2.2.1]heptane core and incorporation of morpholino glycone; previously reported in sordarin derivatives with broad-spectrum antifungal activity (Wu and Dockendorff 2019). Generated novel derivatives as isomeric mixtures (Fig. 11,** compounds 14–17**) were subjected to antifungal microdilution assays, but no growth inhibition was observed against *C. albicans*, *A. fumigatus*, *C. parapsilosis* and *Paecilomyces variotii* at concentration up to 8 μg mL^−1^ or 4 μg mL^−1^.

As far as we know, despite pharmaceutical industry efforts to develop semisynthetic sordarin analogs, no eEF2 inhibitors have advanced to clinical stages.

Recently, it was discovered that eEF2 can also be inhibited by natural products of the honey mushroom *Armillaria mellea* melleolides (Dörfer et al. 2019). Melleolides represent a structurally diverse group of polyketide-sesquiterpene hybrids exerting potent antifungal effects against *Aspergilli* and various other fungi species. Two identified compounds, dehydroarmillyl orsellinate (DAO) and arnamial (Fig. 11,** compounds 18–19**) containing Δ^2,4^-double bond, crucial for antifungal activity, were reported as potent inhibitors of eEF2 in *Aspergillus* and probably *Candida* strains. DAO caused growth inhibition of *A. nidulans*, *Candida albicans*, *Candida parapsilosis,* and *Candida lusitaniae*, however, *Candida glabrata* and *S. cerevisiae* were unaffected. Interestingly, *S. cerevisiae* and *C. albicans* were sensitive to sordarin, while *A. nidulans* and other tested fungal species were resistant. From these observations, it was suggested that sordarins and melleolides target dissimilar sites in eEF2. Elongation factor 3 is not found in mammalian cells, thus it would make a perfect molecular target for antifungal drugs. In fact, in fungal cells, eEF3 encoded by the *YEF3* gene is essential for translation and cell viability (Dever et al. 2016). Although up to our knowledge, there are no reports of any potential inhibitors targeting fungal eEF3. Taken together, translational elongation factors seem to be a valid source of molecular targets for novel antifungal chemotherapy.

### Glycylpeptide *N*-tetradecanoyltransferase (*N*-Myristoyltransferase)

**Fig. 12:**
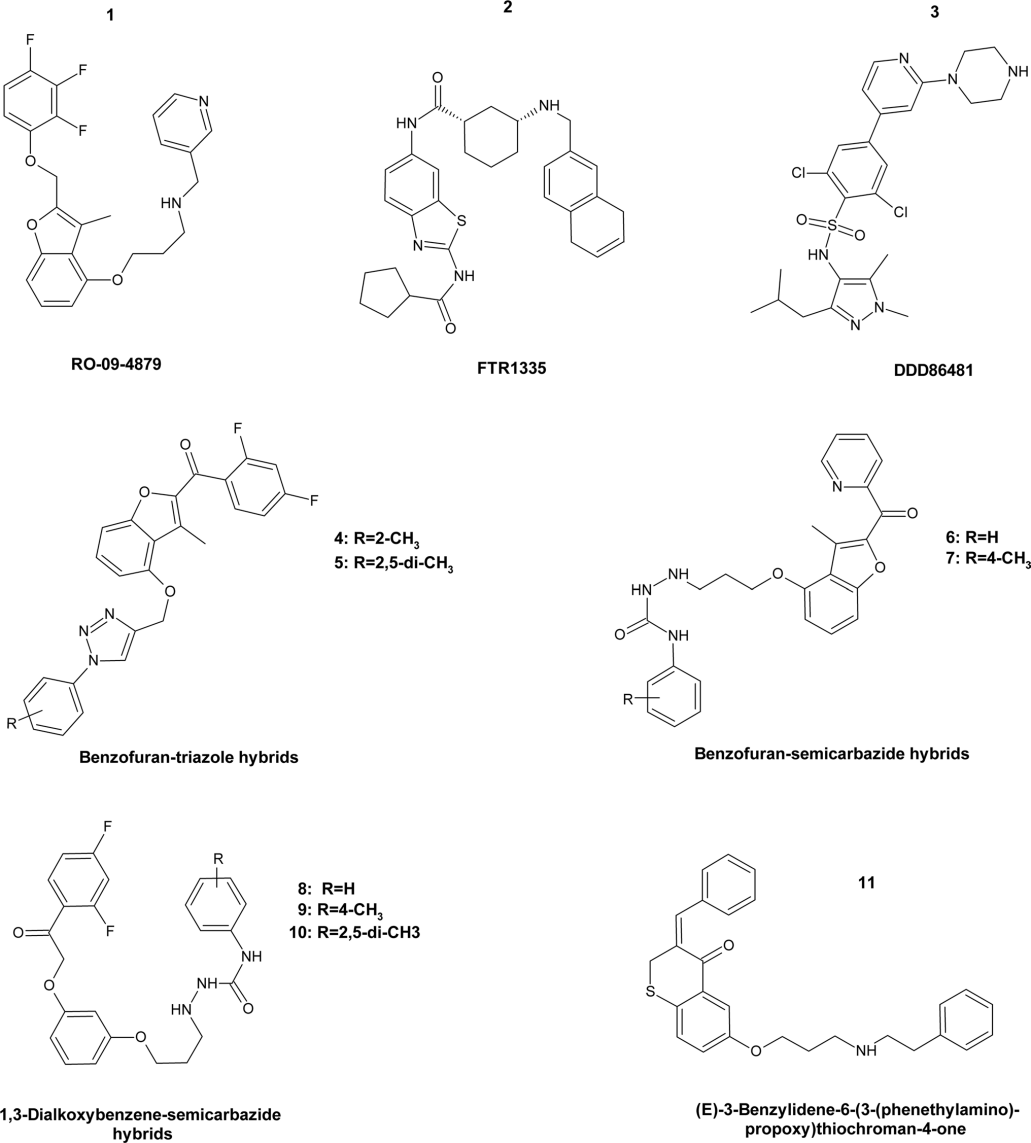
Inhibitors of fungal *N*-myristoyltransferase post-translational modifications. Compound 1 RO-09-4879; compound 2 FTR1335; compound 3 DDD86481; compounds 4–5 Benzofuran-triazole hybrids; compounds 6–7 Benzofuran-semicarbazide hybrids; compounds 8–10 1,3-Dialkoxybenzene-semicarbazide hybrids; compound 11 (E)-3-Benzylidene-6-(3-(phenethylamino)-propoxy)thiochroman-4-one

Glycylpeptide *N*-tetradecanoyltransferase EC 2.3.1.97 known as *N*-myristoyltransferase (NMT) catalyzes co-translational transfer of a myristoyl moiety from myristoyl-CoA (MYA) to the N-terminal glycine of proteins, followed by the removal of the methionine initiator residue. NMT is present in various eukaryotic organisms, however, there are clear differences in peptide substrate specificity between fungal and human enzymes. In fact, fungal NMT was proven to be essential for the growth and viability of *C. albicans* and *C. neoformans* (Lodge et al. 1994; Weinberg et al. 1995), while genetic disruption studies performed on *A. fumigatus* revealed that reduced expression of NMT encoding gene affected cell morphogenesis and cell wall integrity (Fang et al. 2015). *N*-Myristoyltransferase is, therefore, highly investigated as a potential therapeutic target for the treatment of pathogenic fungal infections. Up to now, there are several reports of various NMT inhibitors that include benzofuran derivatives, myristic acid analogues, and peptidomimetics (Liang et al. 2016). The most potent inhibitors reported are RO-09-4879 and FTR1335 (Fig. 12,** compounds 1–2**) displaying high and selective antifungal activity. RO-09-4879 inhibits growth of *C. albicans*, *C. guilliermondii*, *C. tropicalis*, *C. parapsilosis* with MIC values 0.11, 0.44, 0.11, and 0.055 μM, respectively, while FTR1335 inhibits especially *C. albicans*, including fluconazole-resistant isolates with MIC value 0.78 µM (Masubuchi et al. 2003; Ebara et al. 2005). Recently, a screening of inhibitor library led to discovery of a pyrazole sulphonamide derivative DDD86481 (Fig. 12,** compound 3**) that inhibited the *A. fumigatus* NMT enzyme with IC_50_ value 12 nM. Despite highly potent properties, DDD86481 turned out to be a poor fungicidal agent against *A. fumigatus* (MIC 925 μM), however, under partially repressive NMT encoding gene conditions, its fungicidal activity was improved (MIC 7 μM) (Fang et al. 2015). Liang et al. (Liang et al. 2016) designed and synthesized a series of novel benzofuran-triazole hybrids as potential inhibitors of NMT. Designed derivatives possessed a 1,2,3-triazole scaffold that was previously reported in various antifungal agents. Two of the synthesized derivatives (Fig. 12, **compounds 4–5**) exhibited antifungal activity against fluconazole-resistant *T. rubrum* and *C. neoformans*, with MIC in the range of 32–64 µg mL^−1^, which makes them an asset for further research. In a more recent study, novel benzofuran-semicarbazide hybrids and 1,3-dialkoxybenzene-semicarbazide hybrids were identified as potential NMT inhibitors (Xu et al. 2019). Most of the identified compounds displayed in vitro antifungal activity against several fungal strains: *C. albicans, A. fumigatus, T. rubrum, C. krusei* and *C. parapsilosis.* Among all tested compounds, five derivatives (Fig. 12, **compounds 6–10**) were found to inhibit the growth of two fluconazole-resistant *C. albicans* strains isolated from AIDS patients, with MIC values between 2 and 32 µg mL^−1^. Inhibitor of fungal NMT was also found among a series of synthesized novel thiochroman-4-one derivatives (Fig. 12, **compound 11**), which showed antifungal activity against *C. albican*s and *C. neoformans* with MIC of 0.5 µg mL^−1^ and 1 µg mL^−1^, respectively, values similar to those for amphotericin B (Zhong et al. 2017). The structure–activity relationship and molecular docking analysis indicated that the binding model of the found inhibitor to *C. albicans* NMT was consistent with the original NMT ligand R64. Taken together, thiochroman-4-one derivatives might be considered as new promising lead candidates for further development of novel antifungal agents.

**Fig. 13:**
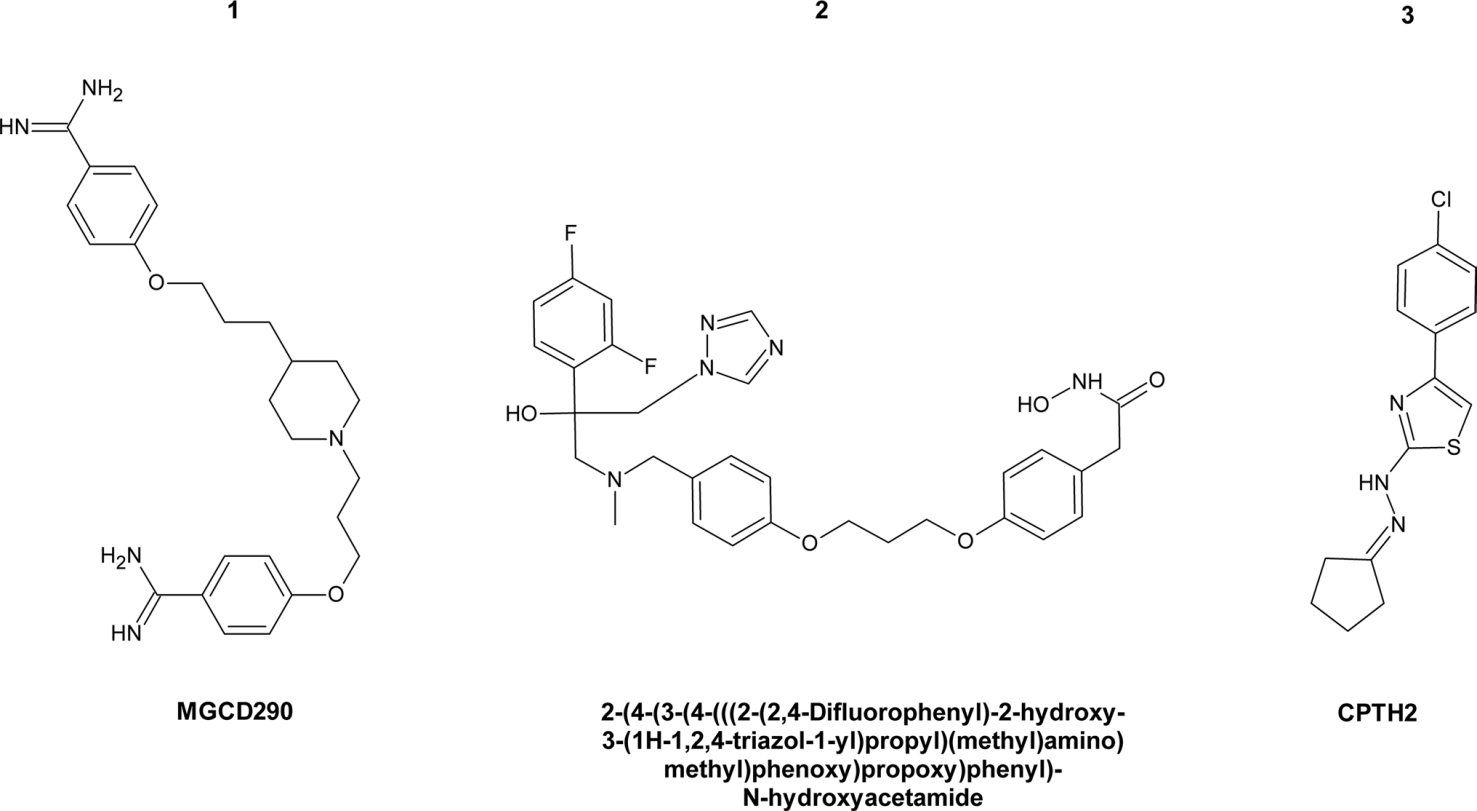
Inhibitors of fungal acetyltransferase enzymes involved in post-translational modifications. Compound 1 MGCD290; compound 2 2-(4-(3-(4-(((2-(2,4-Difluorophenyl)-2-hydroxy-3-(1H-1,2,4-triazol-1-yl)propyl)(methyl)amino)methyl)phenoxy)propoxy)phenyl)-*N*-hydroxyacetamide; compound 3 CPTH2

Majority of eukaryotic proteins undergo reversible post-translational modifications (PTM) that enable them to gain their final activity. These processes include acetylation, methylation, phosphorylation, glycosylation, and ubiquitination of protein molecules (Wassano et al. 2020). Reversible PTMs control various cell processes via regulation of protein function, the most important modification is acetylation of proteins that regulates translation, gene expression, and metabolism (Kuchler et al. 2016). Acetylation is an evolutionarily conserved process that involves acetyltransferase enzymes transmitting the acetyl group from a donor acetyl coenzyme A (CoA) to a suitable acceptor molecule. Histone acetyltransferases EC 2.3.1.48 (HATs) and histone deacetylases EC 3.5.1.98 (HADC) catalyze, respectively, the addition or removal of acetyl groups to or from lysine residues, most importantly in histone proteins, and have been identified as potential drug targets (Wassano et al. 2020). It was revealed that HAT/ HADC activities are required for *C. albicans* virulence. HATs also are involved in the morphogenetic yeast to hyphae transition, drug resistance and biofilm formation (Kuchler et al. 2016). One of the most potent Hos2p histone deacetylase EC 3.5.1.98 (HADC2) inhibitor is MGCD290 (Fig. 13,** compound 1**), which entered phase II of clinical trials in 2013 (Houšť et al. 2020). MGCD290 increases the susceptibility of *Candida* spp*.* and *A. fumigatus* to azole antifungals, however, alone it showed lower antifungal activity (MIC range 4–32 μg mL^−1^) (Pfaller et al. 2009, 2015). Phase II of clinical trials exhibited that this compound did not increase the efficacy of fluconazole therapy in vivo, no further studies in this area are conducted (Gintjee et al. 2020). Recently, Han et al. (2020) discovered the first generation of sterol 14-alpha-demethylase/histone deacetylases CYP51/HDAC EC 1.14.14.154/ EC 3.5.1.98 dual inhibitors, which exhibited potent antifungal activity against azole-resistant candidiasis. One of the designed compounds (Fig. 13,** compound 2**) inhibited the activity of fungal CYP51/HDAC with an IC_50_ value of 0.16 µM. Moreover, it displayed excellent in vitro and in vivo antifungal activity against *C. albicans* and *C. neoformans* with MIC_80_ values of 0.125 µg mL^−1^ and 0.5 µg mL^−1^, respectively, and was active against azole-resistant clinical isolates with MIC_80_ values between 0.25 and 0.5 µg mL^−1^. The inhibitor also prolonged the survival time of mice infected with *C. albicans* and significantly decreased renal fungal burden.

Antifungal activity was also proven for a known Gcn5p histone acetyltransferase EC 2.3.1.48 (HAT) inhibitor, cyclopentylidene-[4-(4-chlorophenyl)thiazol-2-yl)hydrazone (CPTH2) (Fig. 13,** compound 3**) (Chimenti et al. 2009; Tscherner and Kuchler 2019). CPTH2 exhibited selective antifungal activity towards CTG clade fungal species, but especially it had a significant fungicidal effect against *C. albicans.* This compound completely inhibited yeast growth and severely decreased its potential in 50 μM and 25 μM concentrations, respectively. CPTH2 was discovered to protect primary macrophages from *Candida*-mediated death. However, further studies revealed that CPTH2 antifungal effect is independent of direct Gcn5p inhibition, which suggests that Gcn5p might not be the only target of CPTH2, but this requires future verification. Nonetheless, post-translational modifications such as acetylation and deacetylation of proteins provide an interesting source of targets for novel inhibitors that could treat fungal diseases.

### Antifungal peptides targeting protein biosynthesis

There are reports indicating that some peptides may inhibit protein biosynthesis and act as antimicrobial agents (Fernández de Ullivarri et al. 2020). In 2020, Velivelli et al. (2020) showed the fungicidal activity of peptide NCR044 from *Medicago truncatula* (barrelclover). NCR044 inhibits the growth of four economically important fungal pathogens: *F. oxysporum* with IC_50_ value of 0.52 µM, *B. cinerea* with IC_50_ value of 1.55 µM, *F. graminearum* and *F. virguliforme* with IC_50_ value of 1.93 µM and 1.68 µM, respectively. Previously, it was also discovered that nine NCR peptides inhibit the growth of *C. albicans* with MIC values in the range of 10–25 µg mL^−1^, where NCR044 exhibited MIC of 11–12.5 µg mL^−1^. Moreover, all tested peptides were found nontoxic for human cells, however, at higher concentrations, some were reported to affect the proliferation of human cells (Ördögh et al. 2014). These results bring insight into the therapeutic potential of NCR peptides in antifungal chemotherapy. The multistep antifungal mechanism of NCR044 includes an inhibition of translation. This is not the only case of a peptide with such mechanism of action. Méndez et al. (1996) showed that γ-hordothionin plant defensin from *Hordeum vulgare* endosperm, interferes with the chain elongation step of protein biosynthesis in the rabbit reticulocyte system, whereas ω-hordothionin affects the initiation and elongation processes. However, this mechanism of action has never been associated with the antimicrobial activity of these compounds (de Oliveira Carvalho and Moreira Gomes 2012). Several other peptides, plant defensins, with antifungal activity inhibit protein biosynthesis in cell-free extract systems like RBAFP from *Adzuckia angularia*, PBAFP, WCBAFP, vulgarinin from *Phaselus vulgaris*, VaD1 from *Vigna angularis*, SIα_1_ from *Sorghum bicolor* (Chen et al. 2005; Colilla et al. 1990; Osborn et al. 1995; Ye and Ng 2001; Wong and Ng 2005; Wong et al. 2006), but it is not known whether this mechanism of action is significant in vivo in inhibiting fungal growth (van der Weerden and Anderson 2013). This is related to the necessity for peptides to penetrate inside the cell, into the cytoplasm where the cell translation machinery resides. Some plant defensin are able to traverse the fungi plasma membrane, like *Vr*CRP from *Vigna radiate,* which inhibits the growth of *Rhizoctonia solani* (Chen et al. 2002). Whereas antibacterial peptides apidaecins, enter bacterial cell and inhibit protein biosynthesis in vivo in dose-dependent manner (Castle et al. 1999). A similar mechanism has also been proven for animal origin pleurocidin-based and PR-39. It was proven that membrane damaging is not a primal cause of antibacterial activity but rather an inhibition of macromolecular biosynthesis (Boman et al. 1993; Patrzykat et al. 2002).

## Conclusion

Nowadays, there is a growing need for antifungal compounds whose mechanism of action differs completely from drugs used clinically. More than a decade has passed since introducing a new class of antifungal drugs that fight systemic mycoses, whereas there are known cases of fungi resistant to all approved oral drugs. The number of antifungal publications has more than doubled in the last 20 years, which proves the increased interest in this problem and the intensification of research into new compounds fighting mycosis. Finding a compound that kills fungi is easy; the problems that need to be dealt with are the selectivity of antifungals, their toxicity towards host cells, and environmental safety (in the case of compounds used in agriculture). Compounds that inhibit enzymes involved in amino acid biosynthesis, protein biosynthesis and post-translational modification pathways represent a new class of agents that give hope to overcoming these problems and fungal cross-resistance mechanisms. The information provided shows that the branched-chain amino acids, aromatic amino acids, and methionine biosynthetic pathways are particularly important for the virulence of fungal pathogens, however, the target potential of fungal elongation factors is clearly still unexploited. In this paper, we presented compounds with antifungal activity (from the period 2015 to 2020), the molecular targets which are the enzymes involved in the route that leads from amino acid biosynthesis to protein folding and its activation (Table 1). The enzymes described in our review: (1) belong to biosynthetic pathways that do not exist in human cells, or/and (2) their structure is functionally distinct from the human counterpart. We confirmed the potential of the described compounds by describing their effect on fungal strains, but also by analyzing their molecular targets in the cell. We presented research on deletion mutants, their viability, virulence, and the ability to create biofilm. The structures of the described compounds are often based on research conducted in previous years, therefore, we considered it necessary to present the results on these lead compounds. Many enzymatic inhibitors presented in this review exhibit broad-spectrum antifungal activity, MIC values better than commonly used antifungal drugs, low, if any, cytotoxicity against human/mammalian cells and activity against azole-resistant strains. Activity of some has been proven in the infected animal model. Although none of them have yet been accepted for clinical use, efforts are being made to improve their properties or to find novel compounds that satisfy the clinical requirements. The fact that most of them have been presented in recent years clearly indicates that the potential for their further expansion is apparently emerging.

**Table 1:** Selected antifungal compounds active against human pathogens (all mentioned potential antifungals are summarized in the Table 2, supplementary material)

Compound		Molecular target		Pathogen	MIC_90_		Characteristic	
**Aspartate family amino acid biosynthesis pathway**								
RI-331 (HONV)^1,2^ (lead compound)		Homoserine dehydrogenase EC 1.1.1.3		*C. albicans* *C. glabrata* *C. kefyr* *C. tropicalis* *A. fumigates* *C. neoformans* *C. parapsilosis*	100–400 μg mL^−1^ 50–200 μg mL^−1^ 6.25–12.5 μg mL^−1^ 25–> 400 μg mL^−1^ Not active* Not active* Not active*		Well tolerated orally in mice and rats	
**Branched-chain amino acids biosynthesis**								
Ethoxysulfuron^3,4,5,6^ (lead compound)		Acetolactate synthase EC 2.2.1.6		*C. albicans* *C. parapsilosis* *S. cerevisiae*	0.625–2.5 μg mL^−1^ 1.25 μg mL^−1^ 2–5 μg mL^−1^		Relatively low toxicity There is no homolog of this acetolactate synthase in human cells	
**Aromatic amino acid biosynthesis pathway**								
CP1^7^		Chorismate synthase EC 4.2.3.5		*P. brasiliensis* *P. lutzii*	2–16 µg mL^−1^ 16–32 µg mL^−1^		Reducing fungal burden in the lungs and inflammatory response in a mouse infection model Non-cytotoxic against human cell lines	
**Aminoacyl-tRNA synthetases**								
BN-3b^8,9^		ThrRS? EC 6.1.1.3		*A. fumigatus* *C. albicans* *C. parapsilosis*	50 µg mL^−1^ 50 µg mL^−1^ 12.5 µg mL^−1^		Severely damaged *C. albicans* cell membrane and inhibited the hyphal formation	
**Elongation factors**								
R-135853^10^		eEF2 EC 2.7.11.20		*C. albicans* *C. glabrata* *C. tropicalis* *C. parapsilosis* *C. guilliermondii* *C. neoformans* *A. fumigatus* *A. flavus*	0.016–0.03 µg mL^−1^ 0.5 µg mL^−1^ 0.06 µg mL^−1^ 64 µg mL^−1^ 0.5 µg mL^−1^ 0.12 µg mL^−1^ Not active* Not active*		Effective in *C. albicans* infected mice model	
**Glycylpeptide N-tetradecanoyltransferase**								
RO-09-4879^11^		Glycylpeptide *N*-tetradecanoyl transferase EC 2.3.1.97		*C. albicans* *C. parapsilosis* *C. tropicalis* *A. fumigatus* *A. flavus* *C. glabrata* *C. neoformans* *C. krusei*	0.1–6.25 μM 0.05 μM 3.13 μM Not active* Not active* Not active* Not active* Not active*		Active against *C. albicans* AR** isolates	
**Post-translational modifications**								
MGCD290^12^		Histone deacetylase 2 EC 3.5.1.98		*C. albicans* *C. glabrata* *C. krusei* *C. neoformans* *A. fumigatus* *A. flavus* *A. niger* *A. terreus* *Fusarium* spp.	0.5–15 µg mL^−1^ 0.5–4 µg mL^−1^ 2–8 µg mL^−1^ 0.5–4 µg mL^−1^ 8–32 µg mL^−1^ Not active* Not active* Not active* Not active*		Increases the susceptibility to azole antifungals in vitro, however alone it showed lower antifungal activity (MIC range 4–32 μg mL^−1^ Did not increase the efficacy of fluconazole therapy in vivo	
**Antifungal peptides**								
NCR044^13^				*C. albicans*	11–12.5 µg mL^−1^		Nontoxic for human cells—at higher concentrations affect proliferation	

*Inactive in the range of tested concentrations;

**AR azole resistance

^1^Yamaguchi et al. (1988); ^2^Yamaki et al. (1990); ^3^Garcia et al. (2018); ^4^Lee et al. (2013); ^5^Wu et al. (2019); ^6^Zawahir et al. (2009); ^7^Rodrigues-Vendramini et al. (2019); ^8^Hu et al. (2018); ^9^Su et al. (2020); ^10^Kamai et al. (2005); ^11^Ebara et al. (2005); ^12^Houšť et al. (2020); ^13^Ördögh et al. (2014)

Could blocking the enzymes involved in amino acid biosynthesis, protein biosynthesis and post-translational modification pathways be an effective strategy in antifungal therapy?

Using enzymes involved in the amino acid biosynthetic pathway as molecular targets in antifungal therapy is controversial; in some cases, auxotrophs can take the missing amino acid from the environment, eliminating the negative effects resulting from blocking the functioning of enzymes. During the infection, specific niches with different content of amino acids or nitrogen sources are penetrated, which may affect the effectiveness of the compound depending on the site of infection. Moreover, fungi can secrete proteases that enable them to release an amino acid from the host protein. However, these are just some assumptions that would need to be confronted with a particular fungal strain and a molecular target. In our opinion, compounds whose molecular targets are enzymes participating in the pathways described in this review show great potential. *First*, because sufficient number of studies show that the enzymes involved in these pathways are essential for fungi vitality and virulence. Disrupted mutants exhibit a defect in hyphal morphogenesis, biofilm growth, higher sensitivity to antifungals, and reduced virulence in animal-infected model, which may be related to the *second point*; because during infection, the microorganism’s need for amino acids increases due to specific fungal processes like: morphological transition, adhesion, the formation of biofilms, phenotypic switching, stress response machineries associated with the overproduction of numerous proteins. And *third*, significant differences in the structure and functioning between fungal enzymes and relevant enzymes in the human body give hope for the development of a selective antifungal inhibitor, which simultaneously increases the likelihood of obtaining a low-toxic drug. These considerations are confirmed by the low toxicity to human/mammalian cells of the presented compounds.

## Supplementary Information

Below is the link to the electronic supplementary material.

Supplementary file1 (PDF 1027 KB)

## Data Availability

Not applicable.
