# Supplementary material for: Molecular targets for antifungals in amino acid and protein biosynthetic pathways
Source: Amino Acids. 2021 Jun 3;53(7):961–91. doi: 10.1007/s00726-021-03007-6 (PMC8241756; doi:10.1007/s00726-021-03007-6)
Supplement: Supplementary file 1 — Supplementary file1 (PDF 1027 KB) [file 726_2021_3007_MOESM1_ESM.pdf]

SUPPLEMENTARY METERIAL

Molecular targets for antifungals in amino acid and protein biosynthetic pathways

Aleksandra Kuplińska[1], Kamila Rząd[1*]

[1]Department of Pharmaceutical Technology and Biochemistry, Gdańsk University of Technology, Gdańsk, Poland

*** Correspondence:**
Kamila Rząd
kamrzad@pg.edu.pl
ORCID: 0000-0002-6209-6082

Department of Pharmaceutical Technology and Biochemistry,
Gdańsk University of Technology, 11/12 Narutowicza Str.,
80-233 Gdańsk, Poland

Table 2 Potential antifungals: inhibitors of enzymes participating in amino acids and protein biosynthetic pathways

Compound	Structure	Figure	Molecular target	Pathogen	Antifungal activity	Characteristic
**Aspartate family amino acid biosynthesis pathway**						
2-Chloro-3-metoxy-1,4,-naphtoquinone[1]	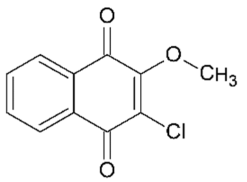	**2.1**	Aspartate semialdehyde dehydrogenase<br>EC 1.2.1.11	*B. dermatitidis*<br>*C. albicans*<br>*C. neoformans*<br>*A. fumigatus*	Ki=2.2 µM<br>Ki=1.6 µM<br>Ki=2.5 µM<br>Ki=0.88 µM	● There is no homolog of the target enzyme in human cells
RI-331 (HONV)<br>(lead compound)[2, 3]	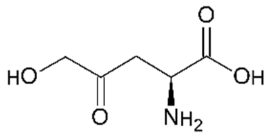	**2.2**	Homoserine dehydrogenase<br>EC 1.1.1.3	*C. albicans*<br>*C. glabrata*<br>*C. kefyr*<br>*C. tropicalis*<br>*A. fumigatus*<br>*C. neoformans*<br>*C. parapsilosis*	MIC90=100-400 µg mL^{-1}<br>MIC90=50-200 µg mL^{-1}<br>MIC90=6.25 – 12.5 µg mL^{-1}<br>MIC90=25 - >400 µg mL^{-1}<br>not active*<br>not active*<br>not active*	● Well tolerated orally in mice and rats<br>● There is no homolog of the target enzyme in human cells

Leu-HONV, Nva-HONV (derivative of HONV) [4]	Leu-HONV<br><br>Nva-HONV	-	Homoserine dehydrogenase<br>EC 1.1.1.3	*C. albicans*<br>*C. glabrata*<br>*C. krusei*<br>*C. parapsilosis*<br>*C. tropicalis*<br>*C. albicans* PRT-OTP	MIC90=32 - 64 μg mL^{-1}<br>MIC90=32 - 64 μg mL^{-1}<br>MIC90=32 - 128 μg mL^{-1}<br>MIC90=128 μg mL^{-1}<br>MIC90=32 - 64 μg mL^{-1}<br>not active*	● Transported to *C. albicans* cells by the di-tripeptide permeases.<br>● HONV is well tolerated orally in mice and rats<br>● There is no homolog of the target enzyme in human cells
HS9[5]	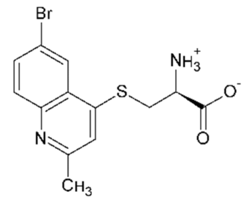	**2.3**	Homoserine dehydrogenase<br>EC 1.1.1.3	*P. brasiliensis*	MIC90=8 μg mL^{-1}	● Low cytotoxicity against human cell lines<br>● The most active inhibitor of *P. brasiliensis* Hom6p<br>● There is no homolog of the target enzyme in human cells
1-Carbohydrazinyl-4-methoxy-1-naphthalene[6]	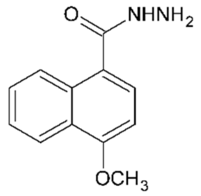	**2.4**	Homoserine dehydrogenase<br>EC 1.1.1.3	*C. albicans*<br>*C. glabrata*<br>*C. parapsilosis*<br>*P. brasiliensis*<br>*P. lutzii*<br>*C. krusei*<br>*C. neoformans*<br>*C. tropicalis*	MIC90=256 μg mL^{-1}<br>MIC90=128 μg mL^{-1}<br>MIC90=128 μg mL^{-1}<br>MIC90=8 - 32 μg mL^{-1}<br>MIC90=16 - 32 μg mL^{-1}<br>not active*<br>not active*<br>not active*	● Non-cytotoxic against monkey kidney and murine macrophage cells<br>● Synergistic antifungal effect with amphotericin B<br>● There is no homolog of the target enzyme in human cells
Rhizocticin A[7]	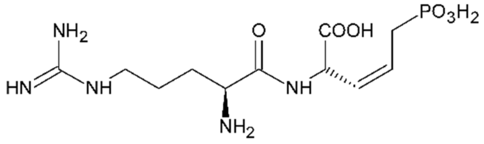	**2.5**	Threonine synthase<br>EC 4.2.3.1	*Aseodesmis sphaerospora*<br>*Basidiobolus microsporus*<br>*Nematospora coryli*<br>*Rhizoctonia solani*<br>*S. cerevisiae*<br>*S. pombe*<br>*Y. lipolytica*<br>*Mierosporum gypseum*<br>*Paecilomyces variotii*<br>*Rhizomucor miehei*<br>*Trichophyton erinaceid*	MIC90=3.5 μg mL^{-1}<br>MIC90=3.5 μg mL^{-1}<br>MIC90=3.5 μg mL^{-1}<br>MIC90=3.5 μg mL^{-1}<br>MIC90=0.35 μg mL^{-1}<br>MIC90=0.35 μg mL^{-1}<br>MIC90=0.35 μg mL^{-1}<br>MIC90>3.5 μg mL^{-1}<br>MIC90>3.5 μg mL^{-1}<br>MIC90>3.5 μg mL^{-1}<br>MIC90>3.5 μg mL^{-1}	● Transported to the fungal cells by oligopeptide transport system
**L-Methionine biosynthesis pathway**						
Bafilomycin C1[8, 9]	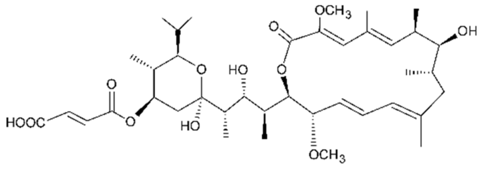	**4.1**	Not proven	*C. albicans*<br>*C. neoformans*<br>*C. parapsilosis*	MIC90=1.56 μg mL-1<br>MIC90=1.56 μg mL-1<br>MIC90=1.56 μg mL-1	● 2.7-Fold down-regulation of expressions methionine synthase EC 2.1.1.13<br>● High cytotoxicity against human cell lines

Cyprodinil[10]	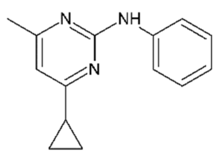	**4.2**	Enzymes involved in methionine biosynthesis pathway?	*S. sclerotiorum*	MIC90< 2 μg mL^{-1}<br>EC50=0.06–0.82 μg mL^{-1} (mycelium growth rate method)	● Inhibits methionine biosynthesis and suppresses cystine and cysteine biosynthesis
(L,L)-2,7,-Bis-(hydrazino)-1,8-octanedioic acid[11, 12]	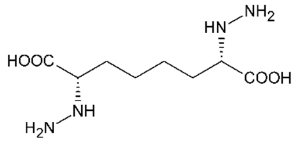	**4.3**	Cystathionine-β-synthase<br>EC 4.2.1.22	*S. cerevisiae*	Not tested against pathogens	● Enzyme-inhibitor modelling study<br>● Proposed as a therapeutic agent for stroke treatment
**Branched-chain amino acids biosynthesis**						
Chlorimuron ethyl[13]<br>(lead compound)	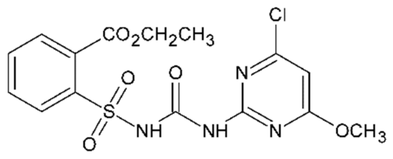	**6.1**	Acetolactate synthase<br>EC 2.2.1.6	*C. albicans*<br>*C. parapsilosis*<br>*C. glabrata*<br>*C. krusei*<br>*C. tropicalis*<br>*C. neoformans*<br>*S. cerevisiae*	MIC50=0.03 μg mL^{-1}<br>MIC50=0.003 μg mL^{-1}<br>MIC50=0.005 μg mL^{-1}<br>MIC50=0.430 μg mL^{-1}<br>MIC50=0.550 μg mL^{-1}<br>MIC50=2.750 μg mL^{-1}<br>MIC50=0.008 μg mL^{-1}	● Reduces overall mortality rates of C. albicans infected mice, by clearing pathogenic fungal burdening of lungs, liver and spleen<br>● Synergistic antifungal effect with itraconazole<br>● Low cytotoxicity against human cell lines<br>● There is no homolog of the target enzyme in human cells
Ethoxysulfuron[13, 17, 18, 19]<br>(lead compound)	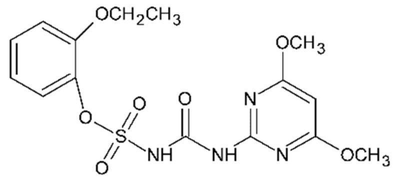	**6.2**	Acetolactate synthase<br>EC 2.2.1.6	*C. albicans*<br>*C. parapsilosis*<br>*S. cerevisiae*	MIC90=0.625-2.5 μg mL^{-1}<br>MIC90=1.25 μg mL^{-1}<br>MIC90=2-5 μg mL^{-1}	● Relatively low toxicity<br>● There is no homolog of the target enzyme in human cells
2-Bromo-N-[(4,6-dimethoxypyrimidin-2-yl)carbamoyl]-5-vinylbenzenesulfonamide [14, 15]<br><br>(derivative of chlorimuron ethyl)	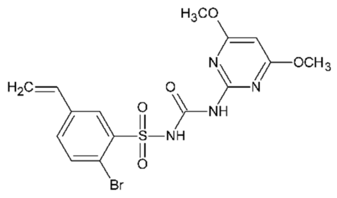	**6.3**	Acetolactate synthase<br>EC 2.2.1.6	*Ceratobasidium cornigerum*<br><br>*Alternaria solani*<br>*Cercospora arachidicola;*<br>*Colletotrichum lagenarium*<br>*Physalospora piricola*<br>*Sclerotinia sclerotiorum*	EC_{50}=4.54 μg mL^{-1} (mycelium growth rate method)<br>50-90% inhibitory rate at 50 μg mL^{-1} (mycelium growth rate method)	● Derivatives of sulfosulfuron exhibit low toxicity in mammals<br>● There is no homolog of the target enzyme in human cells

Sodium[(4,6-dimethoxypyrimidin-2-yl)carbamoyl]{[2-(ethoxycarbonyl)phenoxy] sulfonyl}anide[20, 21]<br><br>(derivative of ethoxysulfuron)	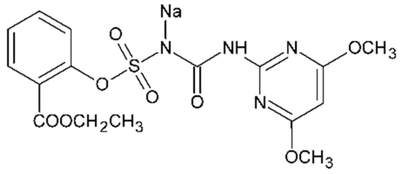	**6.4**	Acetolactate synthase<br>EC 2.2.1.6	*C. albicans*<br>*C. parapsilosis*<br>*S. cerevisiae*	MIC_{90}=1.25 - 2.5 μg mL^{-1}<br>MIC_{90}=1.25 μg mL^{-1}<br>MIC_{90}=2.5 μg mL^{-1}	● Therapeutic effects against *C. albicans* at the nematode model<br>● Derivatives of sulfosulfuron exhibit low toxicity in mammals<br>● There is no homolog of the target enzyme in human cells
Sulfonylurea derivatives containing arylpyrimidine moieties[16, 13]<br><br>(derivative of chlorimuron ethyl)	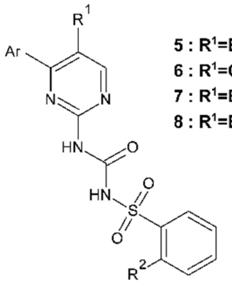<br>**5 : R^1=Br, R^2=NO_2, Ar=methylphenyl**<br>**6 : R^1=Cl, R^2=NO_2, Ar=thiophenyl**<br>**7 : R^1=Br, R^2=NO_2, Ar=thiophenyl**<br>**8 : R^1=Br, R^2=CO_2CH_3, Ar=thiophenyl**	**6.5-8**	Acetolactate synthase<br>EC 2.2.1.6	 *Cercospora arachidicola*<br>*Corticium gramineum*<br>*Fusarium moniliforme*<br>*Fusarium omysporum*<br>*Gibberella zeae*<br>*Helminthosporium maydis*<br>*Physalospora piricola*<br>*Phytophthora infestans*<br>*Rhizoctonia solani*<br>*Sclerotinia sclerotiorum*	Inhibitory rate at 50 μg mL^{-1}:<br>40-88%<br>76-95%<br>83-92%<br>84-92%<br>83-94%<br>53-86%<br>46-96%<br>29-90%<br>72-98%<br>54-83%	● Should be considered as lead compounds for development of acetolactate synthase inhibitors<br>● Derivatives of sulfosulfuron exhibit low toxicity in mammals<br>● There is no homolog of the target enzyme in human cells
3-Substituted phenyl-4-substituted benzylideneamino-1,2,4-triazole derivatives[22]	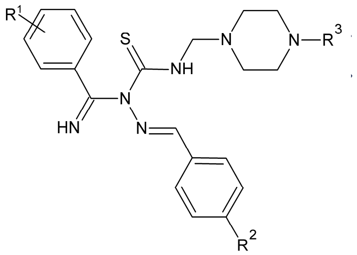<br>**9 :R^1=H, R^2=F, R^3=4-Me-2-pyrimidyl**<br>**10 :R^1=H, R^2=CF_3, R^3=4-Me_2-2-pyrimidyl**<br>**11 :R^1=2-F, R^2=Cl, R^3=4-Me-2-pyrimidyl**<br>**12 :R^1=2-F, R^2=F, R^3=2-pyrimidyl**<br>**13 :R^1=2-F, R^2=F, R^3=4-Me-2-pyrimidyl**<br>**14 :R^1=2-F, R^2=F, R^3=4,6-Me_2-2-pyrimidyl**	**6.9-14**	Ketol-acid reductoisomerase (NADP(+))<br>EC 1.1.1.86	 *Cercospora arachidicola*<br>*Physalospora piricola*<br>*Rhizoctonia cerealis*	inhibitory rate at 50 μg mL^{-1}:<br>14-46%<br>70-80%<br>46-88%	● There is no homolog of the target enzyme in human cells
Myrtenal oxime ester derivatives[23]	**15 :R=CH_3**<br>**16 :R=2,4-ClPh**<br>**17 :R=*m*-CH3Ph**<br>**18 :R=*m*-ClPh**<br>**19 :R=*α*-Furyl**<br>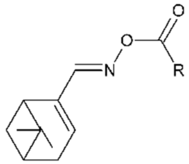	**6.15-19**	Ketol-acid reductoisomerase (NADP(+))<br>EC 1.1.1.86	 *Alternaria solani*<br>*Cercospora arachidicola*<br>*Fusarium cucumerinum*<br>*Fusarium oxysporum*<br>*Gibberella zeae*<br>*Physalospora piricola*	inhibitory rate at 50 μg mL^{-1}:<br>35-66%<br>49-60%<br>21-68%<br>21-68%<br>38-78%<br>53-81%	● There is no homolog of the target enzyme in human cells

L-Cyclopropylalanine[24]	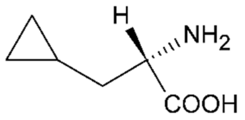	**6.20**	2-Isopropylmalate synthase<br>EC 2.3.3.13?	*C. albicans*<br>*S. cerevisiae*<br><br>*Alternaria alternaria*<br>*Alternaria brassicae*<br>*Alternaria solani*<br>*Ascochyta gossypii*<br>*Cercospora arachidicola*<br>*Cladosporium ladosporium*<br>*Colletotrichum gloeosporioides*<br>*Colletotrichum capsici*<br>*Corynespora cassiicola*<br>*Fusarium graminearum*<br>*Fusarium moniliforme*<br>*Sclerotinia sclerotiorum*	MIC_{90}=44 µM<br>MIC_{90}=19.2 µM<br>inhibitory rate at 50 µg mL^{-1}:<br>58.1%<br>57.4%<br>69.8%<br>86.2%<br>56.8%<br>53.4%<br>74.9%<br>62.1%<br>69%<br>77.2%<br>74.1%<br>100%	● Little, if any toxicity in rats<br>● There is no homolog of the target enzyme in human cells
Hydroxyurea[25]	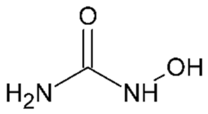	**6.21**	3-Isopropylmalate dehydratase<br>EC 4.2.1.33?	*S. cerevisiae*	Growth assayLeu1 activity was decreased by 3- to 4-fold	● Antiproliferative activity that is widely used in the treatment of chronic myeloid leukemia, sickle cell disease, and AIDS.<br>● Does not alter semi-purified Leu1p activity, even at higher concentrations<br>● There is no homolog of the target enzyme in human cells
**Aromatic amino acid biosynthesis pathway**						
Glyphosate[26]	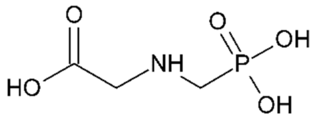	**8.1**	Plant 3-phosphoshikimate 1-carboxyvinyl transferase<br>EC 2.5.1.19,<br><br>an orthologue of yeast Aro1p??	*S. cerevisiae*	Growth assay analysis : affects the growth of yeast strains, but not all at the same rate	● There is no homolog of the target enzyme in human cells
CP1[27]	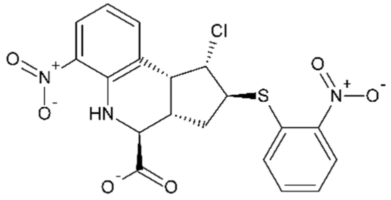	**8.2**	Chorismate synthase<br>EC 4.2.3.5	*P. brasiliensis*<br>*P. lutzii*	MIC_{90}=2-16 µg mL^{-1}<br>MIC_{90}=16-32 µg mL^{-1}	● Reduces fungal burden in the lungs and inflammatory response in a mouse infection model<br>● Non-cytotoxic against human cell lines<br>● There is no homolog of the target enzyme in human cells

CaCS02, CS8[28]	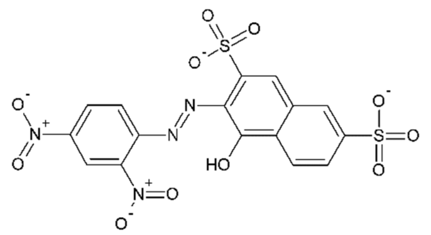	**8.3**	Chorismate synthase<br>EC 4.2.3.5	*P. brasiliensis*<br>*C. albicans*	$MICs_{90}$=32-512 μg mL^{-1}<br>not active*	● Non-cytotoxic against mammalian cell lines<br>● Synergistic antifungal effect with amphotericin B<br>● There is no homolog of the target enzyme in human cells
Β-Sitosterol[29]	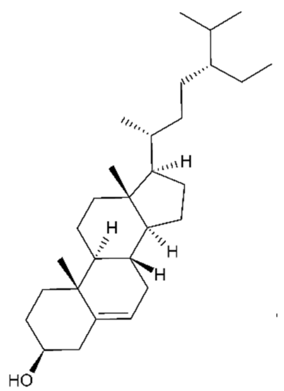	**8.5**	Chorismate synthase<br>EC 4.2.3.5	*A. arborescens*	Inhibitory rate at 500 μg mL^{-1}: 3.5-23%	● Non-toxic, eco-friendly compound<br>● There is no homolog of the target enzyme in human cells
Phytosterols derivatives[29]	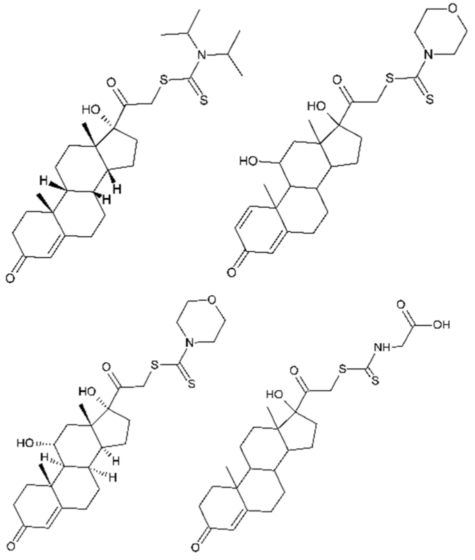	**8.6-10**	Chorismate synthase<br>EC 4.2.3.5		Not tested against pathogens<br>Molecular dynamics simulations of enzyme-inhibitor complexes	● There is no homolog of the target enzyme in human cells

	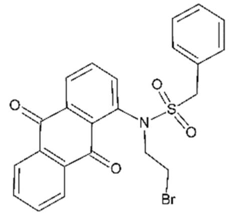					
(S) Abscisic acid[30]	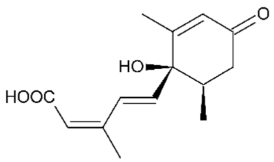	**8.11**	Chorismate mutase<br>EC 5.4.99.5	*A. niger*<br>*C. albicans*<br>*C. parapsilosis*<br>*T. rubrum*	MIC_{90}=125 µg mL^{-1}<br>MIC_{90}=125 µg mL^{-1}<br>MIC_{90}=62.5 µg mL^{-1}<br>MIC_{90}=62.5 µg mL^{-1}	● The inhibition rate was comparable to natural allosteric inhibitor – tyrosine<br>● There is no homolog of the target enzyme in human cells
2-(1-Benzoyl-1H-benzo[d] imidazol-2-ylthio)-N-substituted phenylacetamides derivatives[31]	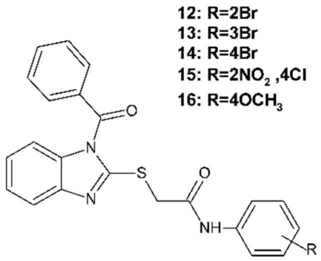	**8.12-16**	Chorismate mutase<br>EC 5.4.99.5?	*A. niger*<br>*C. albicans*	MIC_{90}=0.027 - 0.030 µg mL^{-1}<br>MIC_{90}=0.013 - 0.027 µg mL^{-1}	● There is no homolog of the target enzyme in human cells
5-Fluoroanthranilic acid[32]	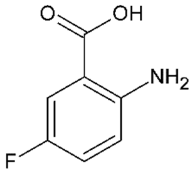	**8.17**	Anthranilate phosphoribosyl transferase<br>EC 2.4.2.18	*C. neoformans*	Growth assay analysis: complete inhibition in YNB medium supplemented with tryptophan	● Antimetabolite<br>● Disturbs cell growth by generating toxic tryptophan analogues<br>● There is no homolog of the target enzyme in human cells
DON[32]	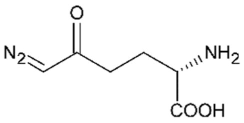	**8.18**	Anthranilate synthase<br>EC 4.1.3.27	*C. neoformans*<br>*C. gatti*	MIC_{90}=62.5 - 125 µM<br>MIC_{90}=125 - 500 µM	● There is no homolog of the target enzyme in human cells
Phosphatidylethanolamine Kdo2-Lipid A[33]	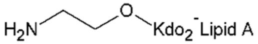	**8.19**	Tryptophan synthase<br>EC 4.2.1.20?		Not tested against pathogens<br>Molecular docking study	● There is no homolog of the target enzyme in human cells

Aminoacyl-tRNA synthetases						
Tavaborole[34, 35, 36, 37]	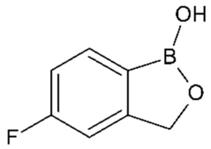	**10.1**	Leucyl-tRNA^{Leu} synthetase<br>EC 6.1.1.4	*Aspergillus spp.*<br>*Candida spp.*<br>*Trichophyton spp.*<br>*Fusarium spp.*	MIC_{90} = 0.5-16 µg mL^{-1}<br>MIC_{90} = 2->16 µg mL^{-1}<br>MIC_{90} = 4-16 µg mL^{-1}<br>MIC_{90} = 8->16 µg mL^{-1}	● Short half-life span used in clinical treatment for topical fungal infections of the nail
Borrelidin[38, 39, 40]	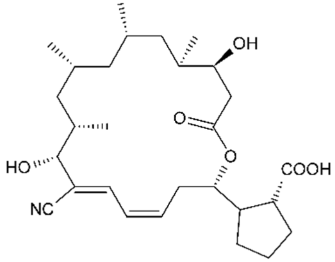	**10.2**	Threonyl-tRNAThr synthetase<br>EC 6.1.1.3	*C. parapsilosis*	MIC_{90} = 50 µg mL^{-1}<br>EC_{50}=0.01-0.1 µg mL^{-1}<br>EC_{50}=0.0056 µg mL^{-1}	● Not used in clinical practice because of its strong cytoxic effect in animal cells
BN-3b[38, 41]	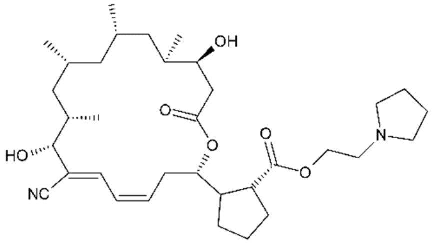	**10.3**	ThrRS?<br>EC 6.1.1.3	*A. alternata*<br>*A. fumigatus*<br>*A. niger*<br>*B. cinerea*<br>*C. albicans*<br>*C. parapsilosis*<br>*C. neoformans*<br>*F. oxysporum*<br>*R. solani*	MIC_{90}=50 µg mL^{-1}<br>MIC_{90}=50 µg mL^{-1}<br>MIC_{90}>100 µg mL^{-1}<br>MIC_{90}=50 µg mL-1<br>MIC_{90}=50 µg mL^{-1}<br>MIC_{90}=12.5 µg mL^{-1}<br>MIC_{90}>100 µg mL^{-1}<br>MIC_{90}=25 µg mL^{-1}<br>MIC_{90}>100 µg mL^{-1}	● Severely damaged *C. albicans* cell membrane and inhibited the hyphal formation
Borrelidin derivatives[38]	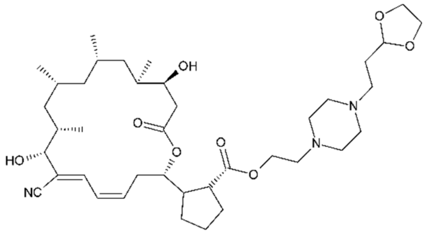<br>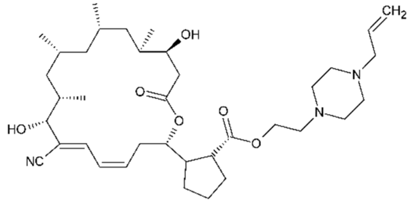	**10.4-5**	Threonyl-tRNAThr synthetase<br>EC 6.1.1.3	*A. fumigatus*<br>*C. albicans*<br>*C. parapsilosis*	MIC_{90} = 12.5 µg mL^{-1}<br>not active<br>not active	● Good selectivity towards fungal cells

**Elongation factors**						
R-135853[42]	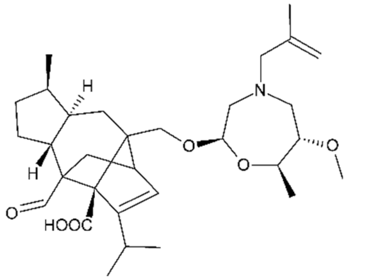	**11.1**	eEF2<br>EC 2.7.11.20	*A. fumigatus*<br>*A. flavus*<br>*C. albicans*<br>*C. glabrata*<br>*C. tropicalis*<br>*C. parapsilosis*<br>*C. guilliermondii*<br>*C. neoformans*	not active*<br>not active*<br>MIC_{90}=0.016 – 0.03 µg mL^{-1}<br>MIC_{90}=0.5 µg mL^{-1}<br>MIC_{90}=0.06 µg mL^{-1}<br>MIC_{90}=64 µg mL^{-1}<br>MIC_{90}=0.5 µg mL^{-1}<br>MIC_{90}=0.12 µg mL^{-1}	● Effective in *C. albicans* infected mice model
Sordaricin derivatives[43]	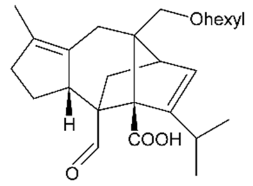<br>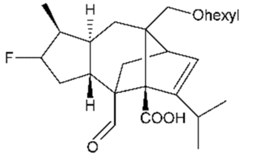	**11.2-3**	eEF2<br>EC 2.7.11.20	*C. albicans*<br>*C. glabrata*	MIC_{90}=0.125- 0.5 µg mL^{-1}<br>MIC_{90}=1- 8 µg mL^{-1}	
Azasordarin derivatives[44]	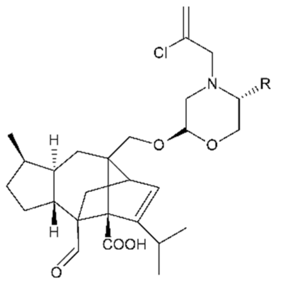<br>**4: R= Me**<br>**5: R= *i*-Pr**<br>**6: R= sprirocyclopentyl**	**11.4-6**	eEF2<br>EC 2.7.11.20	*A. fumigatus*<br>*A. flavus*<br>*C. albicans*<br>*C. glabrata*<br>*C. parapsilosis*<br>*C. neoformans*	MIC_{90}=8 – 32 µg mL^{-1}<br>MIC_{90}=0.125 – 2 µg mL^{-1}<br>MIC_{90}<0.008 - <0.06 µg mL^{-1}<br>MIC_{90}=0.06 – 0.125 µg mL^{-1}<br>MIC_{90}=0.5 – 2 µg mL^{-1}<br>MIC_{90}<0.008 - <0.06 µg mL^{-1}	

Aminopyrrole sordarin derivative[46]	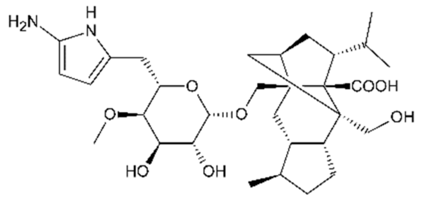	**11.7**	eEF2?<br>EC 2.7.11.20	*S. cerevisiae*	Not tested against pathogens -computer aided simulations of enzyme-inhibitor complexes	
Sordarin diterpene glycosides[45]	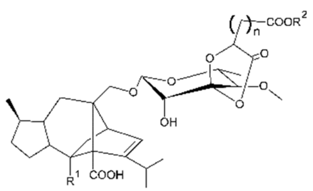	**11.8-13**	eEF2<br>EC 2.7.11.20	*C. albicans*	MIC_{90}=2.9-13 µM	
Simplified azasordarin analogs[47]	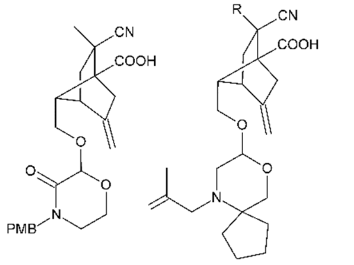	**11.14-17**	eEF2<br>EC 2.7.11.20	*A. fumigatus*<br>*C. albicans*<br>*C. parapsilosis*<br>*P. variotii*	Not active up to 4-8 µg mL^{-1}	
DAO , Arnamial[48]	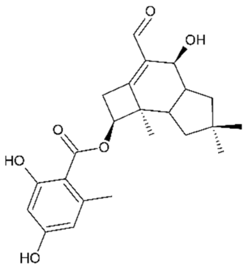<br>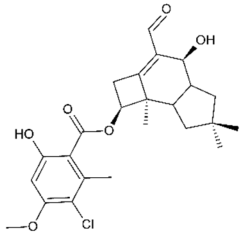	**11.18, 11.19**	eEF2<br>EC 2.7.11.20	*A. nidulans*<br>*C. albicans*<br>*C. parapsilosis*<br>*C. lusitaniae*<br>*C. glabrata*<br>*S. cerevisiae*	Growth assay analysis: affects the growth of strains<br><br>Not active<br>Not active	

**N-Myristoyltransferase**						
RO-09-4879[49]	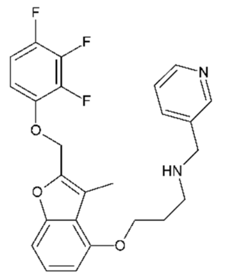	**12.1**	Glycypeptide<br>N-myristoyltransferase<br>EC 2.3.1.97	*C. albicans*<br>*C. neoformans*<br>*C. glabrata*<br>*C. krusei*<br>*C. parapsilosis*<br>*C. tropicalis*<br>*A. fumigatus*<br>*A. flavus*<br>*T. mentagrophytes*<br>*T. rubrum*	MIC_{90}=0.1- 6.25 µM<br>not active*<br>not active*<br>not active*<br>MIC_{90}=0.05 µM<br>MIC_{90}=3.13 µM<br>not active*<br>not active*<br>MIC_{90}=25 µM<br>MIC_{90}=25 µM	● Active against *C. albicans* AR ** isolates
FTR1335[49, 50]	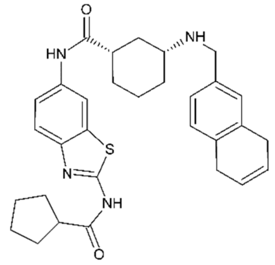	**12.2**	Glycypeptide<br>N-myristoyltransferase<br>EC 2.3.1.97	*C. albicans*<br>*C. neoformans*<br>*C. glabrata*<br>*C. krusei*<br>*C. parapsilosis*<br>*C. tropicalis*<br>*A. fumigatus*<br>*A. flavus*<br>*T. mentagrophytes*<br>*T. rubrum*	MIC_{90}=0.78- 12.5 µM<br>not active*<br>not active*<br>not active*<br>not active*<br>MIC_{90}=3.13 µM<br>not active*<br>not active*<br>not active*<br>not active*	● Active against *C. albicans* AR ** isolates
DDD86481[51]	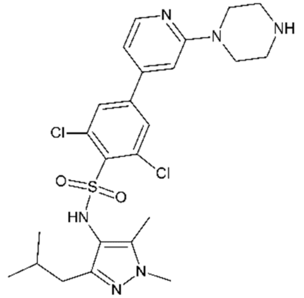	**12.3**	Glycypeptide<br>N-myristoyltransferase<br>EC 2.3.1.97	*A. fumigatus*	MIC_{90}= 925 µM<br>(MIC= 7 µM under partially repressive conditions)	
Benzofuran-triazole hybrids[52]	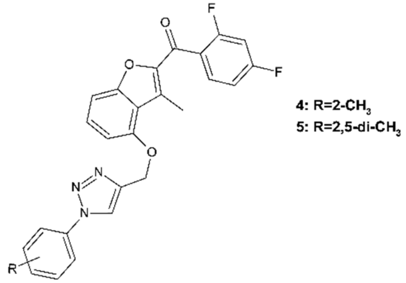	**12.4-5**	Glycypeptide<br>N-myristoyltransferase<br>EC 2.3.1.97	*C. albicans*<br>*C. neoformans*<br>*C. zeylanoides*<br>*R. rubra*<br>*T. rubrum*	MIC_{90}=128 µg mL^{-1}<br>MIC_{90}=32-64 µg mL^{-1}<br>MIC_{90}=64-128 µg mL^{-1}<br>MIC_{90}=128 µg mL^{-1}<br>MIC_{90}=32 µg mL^{-1}	● Active against fluconazole resistant *T. rubrum*, *C. neoformans* AR ** strains

Benzofuran-semicarbazide hybrids,<br>1,3-dialkoxybenzene-semicarbazide hybrids[53]	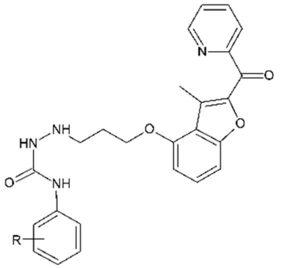<br>6: R=H<br>7: R=4-CH_3<br>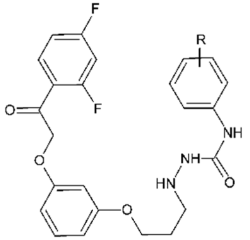<br>8: R=H<br>9: R=4-CH_3<br>10: R=2,5-di-CH3	**12.6-7, 12.8-10**	Glycypeptide<br>N-myristoyltransferase<br>EC 2.3.1.97	*A. fumigatus*<br>*C. albicans*<br>*C. kruseii*<br>*C. parapsilosis*<br>*T. rubrum*	MIC_{90}=2-8 μg mL^{-1}<br>MIC_{90}=2-32 μg mL^{-1}<br>MIC_{90}=4-64 μg mL^{-1}<br>MIC_{90}=0.5>128 μg mL^{-1}<br>MIC_{90}=16-32 μg mL^{-1}	● Active *C. albicans* AR ** strains
(E)-3-Benzylidene-6-(3-(phenethylamino)-propoxy)thiochroman-4-one[54]	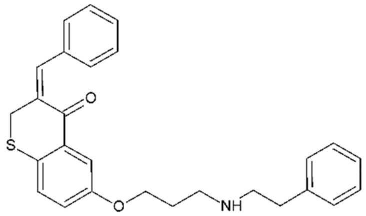	**12.11**	Glycypeptide<br>N-myristoyltransferase<br>EC 2.3.1.97	*A. niger*<br>*C. albicans*<br>*C. neoformans*<br>*M. racemosa*<br>*M. gypseum*<br>*E. floccosum*	not active*<br>MIC_{90}=0.5 μg mL^{-1}<br>MIC_{90}=1 μg mL^{-1}<br>not active*<br>MIC_{90}=8 μg mL^{-1}<br>MIC_{90}=16 μg mL^{-1}	● The antifungal activity was reached to that of fluconazol
**Post-translational modifications**						
MGCD290[55, 56]	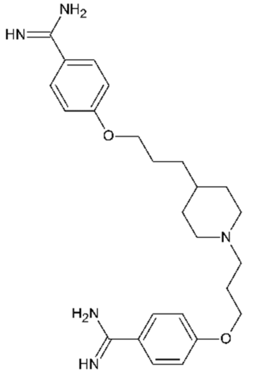	13.1	Histone deacetylase 2<br>EC 3.5.1.98	*C. albicans*<br>*C. glabrata*<br>*C. krusei*<br>*C. neoformans*<br>*Rhodotorula* spp.<br>*Trichosporon* spp.<br>*A.fumigatus*<br>*Mucor* spp.<br>*Rhizopus* spp.<br>*Zygomyces* spp.<br>*S. apiospermum*<br>*A.flavus*<br>*A.niger*<br>*A.terreus*<br>*Fusarium* spp.	MIC_{90}=0.5-15 μg mL^{-1}<br>MIC_{90}=0.5-4 μg mL^{-1}<br>MIC_{90}=2-8 μg mL^{-1}<br>MIC_{90}=0.5-4 μg mL^{-1}<br>MIC_{90}=1-16 μg mL^{-1}<br>MIC_{90}=0.5-8 μg mL^{-1}<br>MIC_{90}=8-32 μg mL^{-1}<br>MIC_{90}=4-32 μg mL^{-1}<br>MIC_{90}=8-32 μg mL^{-1}<br>MIC_{90}=4-32 μg mL^{-1}<br>MIC_{90}=8-32 μg mL^{-1}<br>not active*<br>not active*<br>not active*<br>not active*	● Increases the susceptibility to azole antifungals, however alone it showed lower antifungal activity (MIC_{90} range 4-32 μg mL^{-1})<br>● Did not increase the efficacy of fluconazole therapy *in vivo*

2-(4-(3-(4-(((2-(2,4-Difluorophenyl)-2-2hydroxy-3-(1H-1,2,4-triazol-1-yl)propyl)(methyl)amino)methyl)phenoxy)propoxy)phenyl)-N-hydroxyacetamide[57]	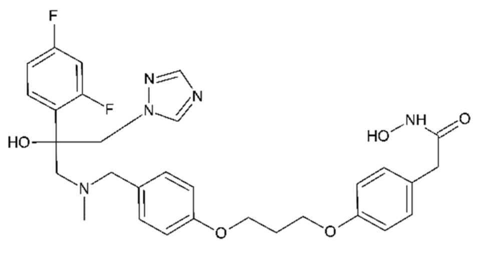	**13.2**	Sterol 14-alpha-demethylase/ histone deacetylases<br>EC 1.14.14.154/ EC 3.5.1.98	*C. albicans*<br>*C. albicans* AR**<br>*C. neoformans* AR**<br>*C. tropicalis* AR**	MIC_{80} =0.125 $\mu g\ mL^{-1}$<br>MIC_{80} =0.25-0.5 $\mu g\ mL^{-1}$<br>MIC_{80} =0.5 $\mu g\ mL^{-1}$<br>MIC_{80} =0.25 $\mu g\ mL^{-1}$	● Inhibitor also prolonged survival time of mice infected with *C. albicans*, and significantly decreased renal fungal burden
CPTH2[58, 59]	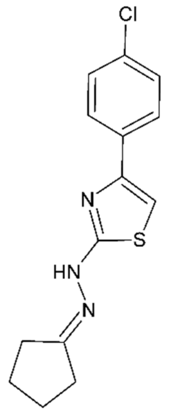	**13.3**	Histone acetyltransferase<br>EC 2.3.1.48	*C. albicans*<br>*C. dubliniensis*<br>*C. guilemondii*<br>*C. glabrata*<br>*C. krusei*<br>*C. kefyr*<br>*C. luistenae*<br>*C. lipopytica*<br>*C. parapsilosis*<br>*C. tropicalis*<br>*S. cerevisae*	Growth assay analysis at 50 μM:<br>Complete inhibition<br>Severe inhibition<br>Complete inhibition<br>not affected<br>complete inhibition<br>not affected<br>some inhibition<br>not affected<br>complete inhibition<br>complete inhibition<br>not affected	● Protects macrophages from Candida-mediated death
**Antifungal peptides**						
NCR044[60, 61]	AFIQLSKPCISDKECSIVKNYRARCRKGYCVRRRIR	-	Protein translation?	*C. albicans*<br>*B. cinerea*<br>*F. oxysporum*<br>*F. graminearum*<br>*F. virguliforme*	MIC=11-12.5 $\mu g\ mL^{-1}$<br>IC_{50}=1.55 μM<br>IC_{50}=0.52 μM<br>IC_{50}=1.93 μM<br>IC_{50}=1.68 μM	● Nontoxic for human cells - at higher concentrations affect proliferation

* inactive in the range of tested concentrations

** AR azole-resistant

[1](Dahal and Viola 2018b); [2](Yamaguchi et al. 1988); [3](Yamaki et al. 1990); [4](Skwarecki et al. 2018); [5](Bueno et al. 2019b); [6](Bagatin et al. 2019); [7](Kugler et al. 1990); [8](Ding et al. 2016); [9](Su H et al. 2018); [10](Hou et al. 2018); [11](Tu et al. 2018); [12](McCune et al. 2016); [13]Garcia et al. 2018); [14](Wei et al. 2016); [15](Healy et al. 2004); [16](Chen et al. 2019b); [17](Lee et al. 2013); [18](Wu et al. 2019); [19](Zawahir et al. 2009); [20](Wu et al. 2019); [21](Garcia et al. 2018); [22](Wang et al. 2017); 23(Lin et al. 2019); [24](Ma et al. 2017); [25](Huang et al. 2016); [26](Rong-Mullins et al. 2017); [27](Rodrigues-Vendramini et al. 2019); [28](Bueno et al. 2019a); [29](Choudhary et al. 2020); [30](Khedr et al. 2018); [31](Yadav et al. 2018); [32](Fernandes et al. 2015); [33](Srivastava et al. 2018); [34](Abastabar et al. 2015); [35](Gupta et al. 2016); [36](Coronado et al. 2015); [37](Markham 2014); [38](Hu et al. 2018); [39](Gao et al. 2012); [40](Wilkinson et al. 2006); [41](Su et al. 2020); [42](Kamai et al. 2005); [43](Regueiro-Ren et al. 2002); [44](Serrano-Wu et al. 2003); [45](Zhang et al. 2019); [46](Chakraborty et al. 2016); [47](Wu and Dockendorff 2019); [48](Dörfer et al. 2019); [49](Ebara et al. 2005); [50](Masubuchi et al. 2003); [51](Fang et al. 2015); [52](Liang et al. 2016); [53](Xu et al. 2019); [54](Zhong et al. 2017); [55](Pfaller et al. 2009); [56](Houšť et al. 2020); [57](Han et al. 2020); [58](Chimenti et al. 2009); [59](Tscherner and Kuchler 2019); [60](Velivelli et al. 2020); [61](Ördögh et al. 2014)
